# Plasma cell output from germinal centers is regulated by signals from Tfh and stromal cells

**DOI:** 10.1084/jem.20160832

**Published:** 2018-04-02

**Authors:** Yang Zhang, Laura Tech, Laura A. George, Andreas Acs, Russell E. Durrett, Henry Hess, Lucy S.K. Walker, David M. Tarlinton, Anne L. Fletcher, Anja Erika Hauser, Kai-Michael Toellner

**Affiliations:** 1 Institute of Immunology and Immunotherapy, Medical School/IBR, University of Birmingham, Birmingham, England, UK; 2 Deutsches Rheuma-Forschungszentrum Berlin, a Leibniz Institute, Berlin, Germany; 3 Division of Genetics, Department of Biology, Friedrich-Alexander-University Erlangen-Nuremberg, Erlangen, Germany; 4 Institute for Cell and Molecular Biology, University of Texas at Austin, Austin, TX; 5 Translational Innovation Platform, Immunology, Merck KGaA, Darmstadt, Germany; 6 Division of Infection & Immunity, Institute of Immunity & Transplantation, University College London, London, England, UK; 7 The Walter and Eliza Hall Institute of Medical Research, Melbourne, Australia; 8 Charité Universitätsmedizin, Berlin, Germany

## Abstract

Germinal centers (GCs) are the sites where B cells undergo affinity maturation. The regulation of cellular output from the GC is not well understood. Here, we show that from the earliest stages of the GC response, plasmablasts emerge at the GC–T zone interface (GTI). We define two main factors that regulate this process: Tfh-derived IL-21, which supports production of plasmablasts from the GC, and TNFSF13 (APRIL), which is produced by a population of podoplanin^+^ CD157^high^ fibroblastic reticular cells located in the GTI that are also rich in message for IL-6 and chemokines CXCL12, CCL19, and CCL21. Plasmablasts in the GTI express the APRIL receptor TNFRSF13B (TACI), and blocking TACI interactions specifically reduces the numbers of plasmablasts appearing in the GTI. Plasma cells generated in the GTI may provide an early source of affinity-matured antibodies that may neutralize pathogens or provide feedback regulating GC B cell selection.

## Abstract (precis)

Plasmablasts generated in germinal centers (GC) emerge at the GC–T zone interface (GTI). Zhang et al. demonstrate two major regulators of this process: Tfh-derived IL-21 and APRIL produced by CD157^high^ fibroblastic reticular cells located in the GTI.

## Graphical Abstract

**Figure:**
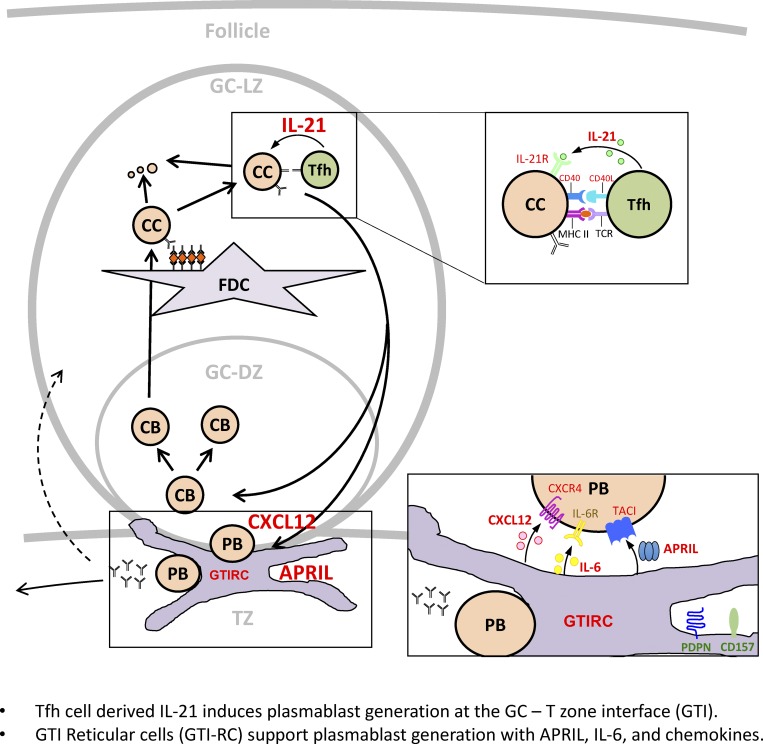

## Introduction

A hallmark of antibody responses to T-dependent antigens is the increase in affinity of antigen-specific antibodies in circulation. Antibody affinity maturation takes place in B cells differentiating in germinal centers (GCs; MacLennan, 1994; Victora and Nussenzweig, 2012). Before the initiation of GCs, some B cells rapidly mature into extrafollicular plasma cells (PCs) that generate an early low-affinity germline-derived antibody (MacLennan et al., 2003). Increases in antibody affinity are easily detectable after secondary immunization (Eisen and Siskind, 1964), but also noticeable during the primary response (Takahashi et al., 1998; Kang et al., 2015). Mutated PCs were found as early as 10 d after primary immunization (Jacob and Kelsoe, 1992; Smith et al., 1997), which is only a few days after the onset of mutational activity in primary GCs (Weiss et al., 1992; Jacob et al., 1993; McHeyzer-Williams et al., 1993). In carrier-primed responses, when T cell help is available immediately, extrafollicular and follicular B cell differentiation happens more rapidly, and mutated PCs are found in the splenic red pulp as early as 2 d after GC formation (Sze et al., 2000). Affinity-increased antibody can appear in blood at the same time (Zhang et al., 2013). Considering mutated GC-derived PCs compete with the initially formed extrafollicular PCs (Sze et al., 2000), this increase in circulating antibody is remarkably fast.

A recent study demonstrated that GCs mature, going through stages of preferential output of memory B cell or long-lived PCs homing to the bone marrow (Weisel et al., 2016). The antibody is not only important for pathogen defense, but it also has a role in regulating B cell selection in the GC by modulating antigen accessibility, shielding antigens from access by lower-affinity B cells (Zhang et al., 2013). For this antibody feedback to happen efficiently, it is critical that GCs produce affinity-matured PC output generating a higher-affinity antibody from an early stage. A recent study showed that the high-affinity antigen interaction of GC B cells triggers PC differentiation, whereas additional undefined signals from T follicular helper (Tfh) cells are necessary to fully induce PC differentiation (Kräutler et al., 2017). In the current study, we set out to test when and where PCs generated from GCs appear locally. We show that this starts from a very early stage of GC development. During the earliest stages of GC differentiation, PCs leave the GC by entering the T zone from the GC dark zone. Defining timing and location of PC output enabled us to identify factors that regulate the appearance of affinity-matured PCs from the GC. We show a role for IL-21, a B cell differentiation factor produced by Tfh cells that is also involved in extrafollicular PC differentiation (Linterman et al., 2010; Zotos et al., 2010; McGuire et al., 2015). We further demonstrate that the GC–T zone interface (GTI) contains a new T zone stromal cell subset producing APRIL, which can support differentiation of PCs in the GTI.

## Results

### Lymphocyte activation and the appearance of GC-derived plasmablasts

The timing and location of plasmablasts emerging in the spleen were tested by immunizing naive mice with sheep red blood cells (SRBCs). i.v. injection of SRBCs induces a synchronized onset of primary T and B lymphocyte activation, leading to extrafollicular plasmablast differentiation and formation of GCs. To follow plasmablast appearance, spleen sections were labeled for the transcription factor IRF4. IRF4 is expressed at low levels in activated B and T cells (Matsuyama et al., 1995; Klein et al., 2006; Sciammas et al., 2006), but is strongly induced as B cells initiate PC differentiation (unpublished data; Klein et al., 2006; Sciammas et al., 2006).

**Figure 1.**
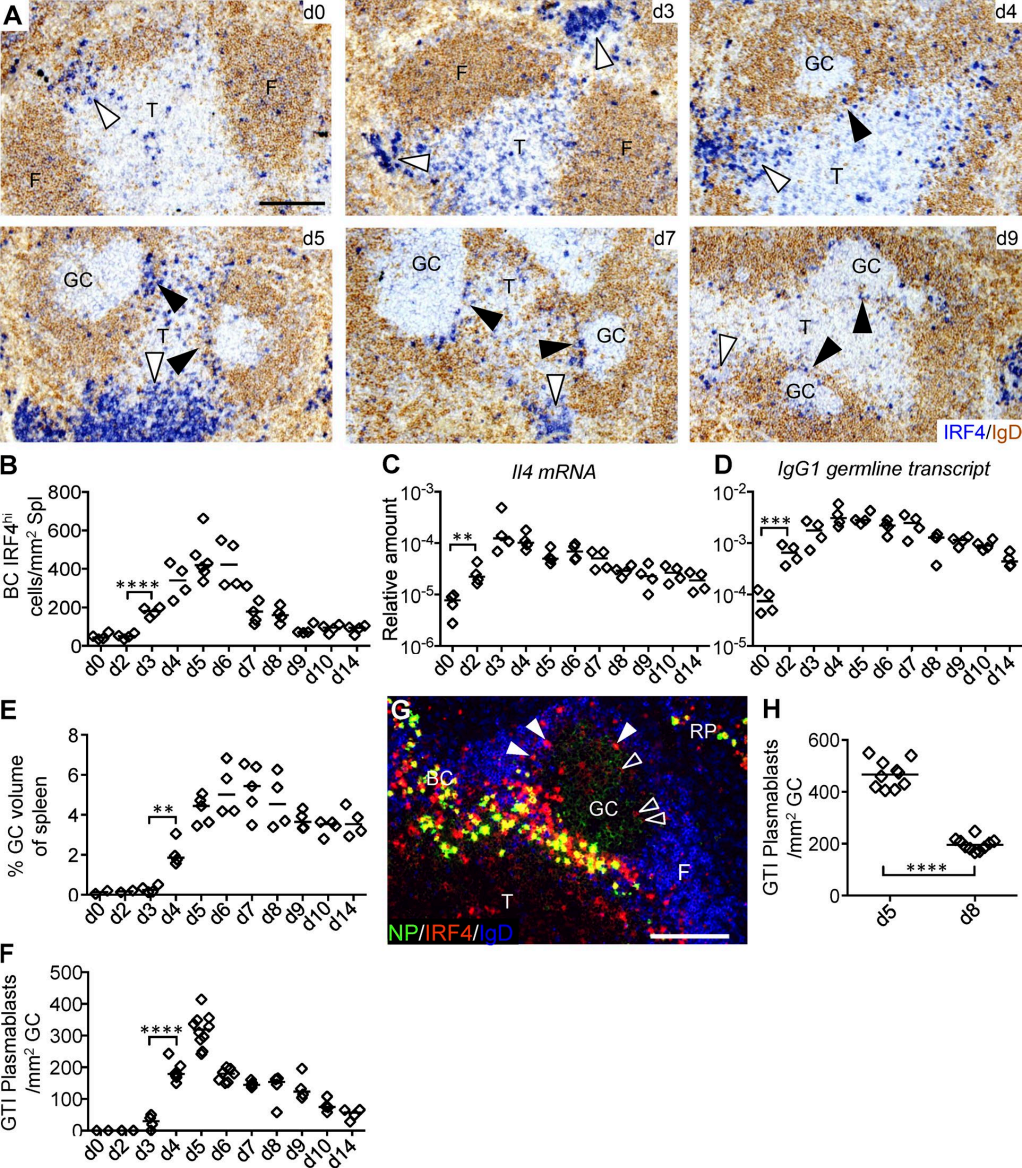
**Appearance of GC-associated PCs during the primary GC response. (A)** C57BL/6 mice were immunized i.v. with SRBCs. Immunohistochemistry for IgD to identify B cell follicles and for IRF4 to identify IRF4^hi^ plasmablasts and PCs in red pulp bridging channels (indicated with open arrowheads) and GTI (closed arrowheads). IRF4^int^ cells in T zone are activated T cells. F, follicle; T, T zone. Bar, 50 µm. **(B)** Number of IRF4^hi^ cells in BCs over time after SRBC immunization. **(C and D)** IL-4 mRNA and germline IgG1 transcripts measured from whole frozen spleen sections. All values are relative to β_2_-microglobulin. **(E)** Splenic GC volume (**, P = 0.0019). **(F)** Number of IRF4^hi^ cells in the GTI per mm^2^ GC area over time after immunization. Data from three independent experiments (two to three mice each time). **(G)** IRF4^hi^ cells in the GTI 5 d after NP-CGG immunization of carrier-primed mice. Immunofluorescence labeling for NP-specific antibody, IRF4, and IgD. Bar, 25 µm. **(H)** IRF4^hi^ cells in the GTI 5 d and 8 d after immunization with NP-CGG in carrier-primed mice. Data from two independent experiments (*n* = 10). Each diamond represents one animal. Nonpaired two-tailed Student’s *t* test. **, P = 0.0091; ***, P = 0.0007; ****, P < 0.0001.

SRBCs induced rapid extrafollicular plasmablast differentiation from day 3 to 5 after immunization (Fig. 1 A). Similar to responses to other antigens (Jacob and Kelsoe, 1992; Toellner et al., 1996, 1998), these appeared in the bridging channels connecting the T zone with the red pulp (Fig. 1 A), but peaked by day 5 (Fig. 1 B). T cell activation, indicated by the significant increase of *Il4* mRNA (Fig. 1 C) and appearance of IRF4^int^ T cells (Fig. 1 A), occurred by day 2 after immunization. A rise in germline IgG1 transcripts suggests that cognate T–B interaction must have happened at the same time (Fig. 1 D).

GCs appeared in a significant amount from day 4 after immunization (Fig. 1 A). Throughout the GC response, IRF4^high^ cells are found at the GTI, suggesting that output of PCs from the GC takes place in this area (Fig. 1 A). Although antibody-forming cells located close to the GC in T zones of lymph nodes have been noticed before (Mohr et al., 2009; Meyer-Hermann et al., 2012), it was surprising to find that these are prevalent during the earliest stages of GC development and peak within 24 h after the emergence of GCs (day 5; Fig. 1 F). Less frequently, IRF4^high^ cells were observed in the periphery of the GC light zone (Fig. 1 A), although this occurs more often at later stages (Angelin-Duclos et al., 2000).

The early onset of PC differentiation at the GTI is not only seen in primary SRBC responses, as challenge of carrier-primed mice with soluble chicken gamma globulin (CGG) coupled to 4-hydroxynitrophenyl (NP) induced even larger numbers of IRF4^high^ PCs at the GTI (Fig. 1, G and H). Again, relative to the size of the GC, appearance of these cells was maximal within 2 d of the appearance of GCs (Fig. 1 H; Toellner et al., 1996). Occasionally, IRF4^int^ B cells could be observed in the GC light zones (Fig. 1 G, open arrowheads).

In summary, these observations are consistent with recent data that GC B cells are selected in the GC light zone through interactions with antigen and Tfh cells (Kräutler et al., 2017), then migrate toward the GC dark zone and GTI, further up-regulating IRF4 expression. At the GTI, they exit the GC as plasmablasts (Meyer-Hermann et al., 2012).

### Characterization of IRF4-expressing cells on the GTI

**Figure 2.**
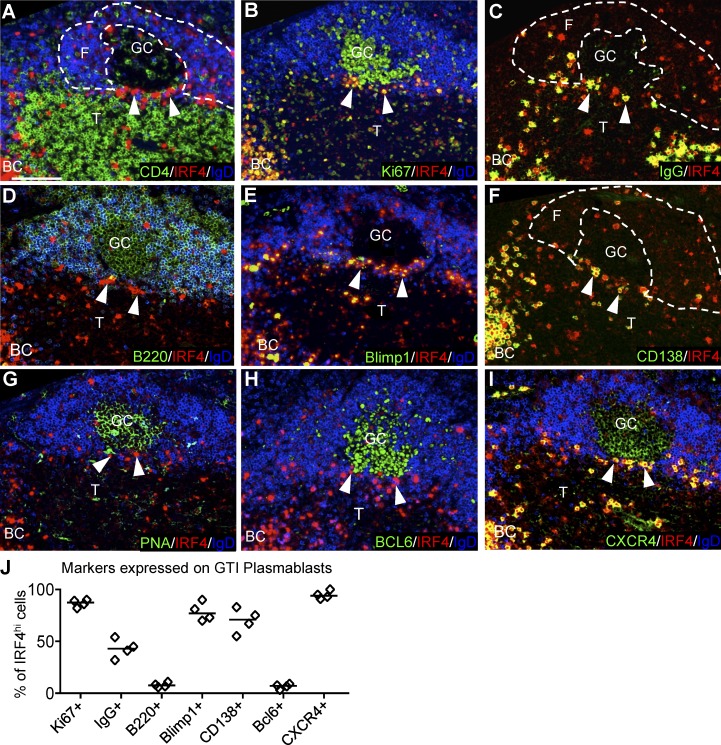
**IRF4hi cells in the GTI express PC-associated antigens**. **(A–I)** Spleens taken from C57BL/6 mice 5 d after immunization with SRBCs (i.v.). Adjacent sections were stained with IRF4, IgD, and various antigens as indicated in the figure. BC, bridging channel; T, T zone; F, follicle. Bar, 50 µm. Dashed lines indicate outlines of B cell follicle and GC. **(J)** Quantification of different markers expressed on IRF4^hi^ cells in the GTI.

To confirm that the IRF4^high^ cells emerging at the GTI are plasmablasts and to test whether these cells still express GC related markers that could be exploited for isolation and characterization, expression of a large range of antigens associated with GC and PC phenotype was tested by immunohistology at the peak of the response, 5 d after SRBC immunization. This shows IRF4^high^ cells appear in a narrow area rich in CD4 T cells, bordering the IgD^−^ GC (Fig. 2 A). IRF4^high^ cells in the GTI were found along a narrow strand of IgD^+^ B cells, identifying this area as the border between the GC-containing follicle and the T zone (Fig. 2 A). IRF4^high^ cells in this area were in close contact with CD4 T cells. Lower numbers of IRF4-expressing cells were found in the light zone of the GC, often in contact with CD4^+^ Tfh cells (Fig. 2 A). Ki-67 staining shows that IRF4^high^ cells in the GTI were still in cell cycle (Fig. 2 B). Furthermore, they accumulated BrdU injected i.p. 2 h before tissue analysis (unpublished data). This identifies them as blasts. Many, but not all, were IgG switched. They expressed Ig as strongly as extrafollicular plasmablasts in the red pulp (Fig. 2 C). Most cells lacked B220 (Fig. 2 D) and expressed Blimp1 (Fig. 2 E) and CD138 (Fig. 2 F), which again is consistent with these cells being plasmablasts. Interestingly, IRF4^high^ cells found in the outer light zone of the GC expressed lower levels of Blimp1 and lower levels of CD138 (Fig. 2, E and F). Because IRF4 is upstream of Blimp1 and differentiation to PCs (Sciammas et al., 2006), this indicates that the IRF4^high^ cells in the outer light zone are an earlier maturation stage than the Blimp1^high^ and CD138^high^ plasmablast in the GTI. Plasmablasts in the GTI were not associated with markers expressed in the GC, including peanut agglutinin binding (Fig. 2 G), BCL6 (Fig. 2 H), or CD21 (not depicted). IRF4^high^ cells strongly expressed CXCR4 (Fig. 2 I), and some of them expressed CCR7 (not depicted), which may aid their migration to the GTI (Bannard et al., 2013).

Collectively, this identifies IRF4^high^ cells in the GTI as plasmablasts (Fig. 2 J). The cells may have been induced to differentiate in the GC light zone and arrive via migration along the GC outer zone (Liu et al., 1992). Expression of CXCR4 and CCR7 may guide their migration toward the GTI (Bannard et al., 2013; Rodda et al., 2015). These chemokine receptors also would have the potential to support further movement of plasmablasts toward splenic bridging channels (Hargreaves et al., 2001). GCs in lymph nodes have a similar GTI that contains plasmablasts that appear to be migrating toward the medulla (Mohr et al., 2009).

### In vivo migration of plasmablasts at the GTI

**Figure 3.**
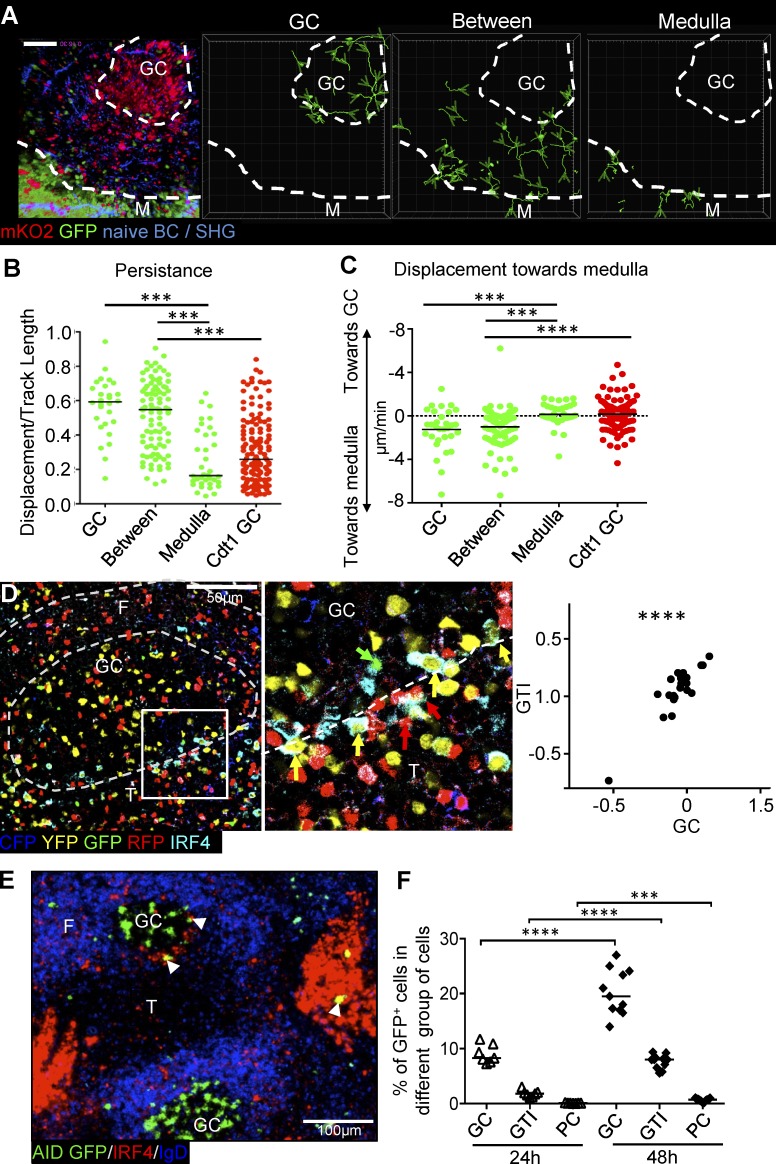
**Dynamics and motility of PCs of different localization during immune response.** NP-specific B cells from B18i/k^−/−^/Blimp1^GFP^/Cdt1^mKO2^ mice were transferred into C57BL6 recipients followed by foot immunization with 10 µg NP-CGG in CFA 24 h later. Popliteal lymph nodes were analyzed 6 d later. **(A)** A 57-µm maximum intensity Z-projection from two-photon microscopy image stacks of a GC and medulla (M) in a live popliteal lymph node. NP-specific transferred B cells and plasmablasts in proliferation are mKO2^+^ and Blimp1-expressing plasmablasts, and PCs are GFP^+^. SHG, second harmonic generation. Bar, 100 µm. Dashed lines indicate the outline of the GC and medulla. Tracks of GFP^+^ cells in GC, area between GC, and medulla and within medullary cords are shown. Track starts are indicated with light green arrowheads. **(B)** Persistence of tracks (track displacement length/track length) of GFP^+^ or mKO2^+^ cells in different areas. Horizontal bar indicates median. **(C)** Displacement of cells moving in up (toward the GC) or down (toward the medulla) direction. Displacement normalized to track observation period. Data merged from three independent experiments (*n* = 3). Each symbol corresponds to one cell. Two-sided Mann-Whitney test. **(D)** Spleen section from Rosa26^confetti^Cre^ERT2^ mouse stained with IRF4. Colored arrows indicate the color of fluorescent protein expressed by IRF4^+^ GTI plasmablasts. Representative pictures are from two independent experiments (*n* = 10). Correlation of first principle component of CFP, GFP, YFP, and RFP expression patterns between GC and corresponding GTI (right). Dashed lines indicate the outline of the GC and B cell follicles. Spearman rank correlation, r = 0.719. **(E)** AID-Cre^ERT2^ × ROSA^mT/mG^ spleen section taken 48 h after tamoxifen induction on day 4. Stained for IRF4 and IgD. White arrowheads indicate GFP^+^ IRF4^high^ cells. **(F)** Quantification of AID-GFP^+^ in each group at 24 h and 48 h after tamoxifen induction. Data merged from three independent experiments (two to three mice each time). Nonpaired two-tailed Student’s *t* test. ***, P = 0.0002; ****, P < 0.0001.

Several strategies were used to more directly show that plasmablasts emerging in the GTI are derived from adjacent GCs. A small number of observations could be made using intravital multiphoton microscopy. Mice received adoptive transfers of NP-specific B cells from B18i/k^−/−^/Prdm1^GFP^/Cdt1^mKO2^ mice. These mice express GFP under the control of the *Prdm1* promoter (Kallies et al., 2004), and express a fast-folding version of monomeric Kusabira Orange (mKO2) during the G1 phase of the cell cycle (Sakaue-Sawano et al., 2008), indicating fate decision toward PC differentiation by GFP, whereas GCs can be identified by mKO2 expression. Plasmablast migration was followed 6 d after foot immunization with NP-CGG in complete Freund’s adjuvant. Observations showed the presence of Blimp1-GFP–expressing cells in the GC and in the zone between the GC and the medulla (Fig. 3 A). Blimp1-expressing cells were found in large numbers in the medulla and in smaller numbers in GCs and the space between the GC and the medulla. Blimp1-expressing cells in the GC preferentially moved toward the GTI, with few traversing into the surrounding space. Similar to earlier observations (Fooksman et al., 2010), Blimp1-expressing cells between the GC and medulla were mobile, whereas cells in the medullary cords were more or less stationary (Fig. 3 B). Analysis of the movement of plasmablasts along the GC–medullary axis showed a slightly higher tendency of net migration toward the medulla (Fig. 3 C).

To test whether cells in the GTI are clonally related to cells in the GC, Confetti mice (Snippert et al., 2010) were induced to randomly express CFP, hrGFP, YFP, or dsRFP and immunized with SRBCs. After 5 d, the distribution of different colors in GCs and adjacent GTIs were observed by microscopy (Fig. 3 D). Comparing the distribution of different colors (Fig. S1) by principal component analysis revealed a positive correlation between GCs and plasmablasts in the adjacent GTI (Fig. 3 D).

To further test whether cells in the GTI originate from the GC, Aicda-CreERT2 mice (Dogan et al., 2009) were crossed onto mTmG mice (Muzumdar et al., 2007) permitting *Aicda*-expressing cells to express GFP after induction of Cre by tamoxifen. Mice were immunized with SRBCs, and Cre was induced 4 d after immunization. 5 and 6 d after immunization, GFP^+^ cells in the GC, GTI, and in extrafollicular foci in bridging channels were analyzed. This showed that 24 h after Cre induction, 9.0% of GC cells and 1.8% of GTI plasmablasts were GFP^+^, whereas PCs in bridging channels were GFP^−^. Another 24 h later, 19% of GC B cells were GFP^+^, and 8.3% of GTI plasmablasts and 1.1% of bridging channel PCs had become GFP^+^.

These observations are consistent with plasmablasts in the GTI being descendants of adjacent GCs in transit toward bridging channel or medullary PC niches (Sze et al., 2000; Mohr et al., 2009) with a 24 h delay between cells expressing *Aicda* in the GC and the first arrival of their descendants in the GTI. These timings correlate very well with earlier observations on germinal center dynamics (Victora et al., 2010).

### Expression of mRNA coding for IL21 in the GC and APRIL in the GTI

To test signals regulating plasmablast differentiation from the GC, sections of spleens taken from carrier CGG–primed mice 5 d after challenge with NP-CGG were separated into the B cell follicle, GC, GTI, extrafollicular PC foci, and T zone using laser microdissection (Zhang et al., 2016). Real time PCR (RT-PCR) for expression of *Cd3e*, *Cd19*, *Pax5*, and several other GC- and PC-associated genes demonstrate that the technique distinguishes these specific splenic microenvironments (Fig. S2). Despite the GTI being quite narrow, which may lead to contamination from neighboring areas, there was 20× lower *Bcl6* and *Aicda* mRNA detected compared with neighboring GCs (Fig. S2).

**Figure 4.**
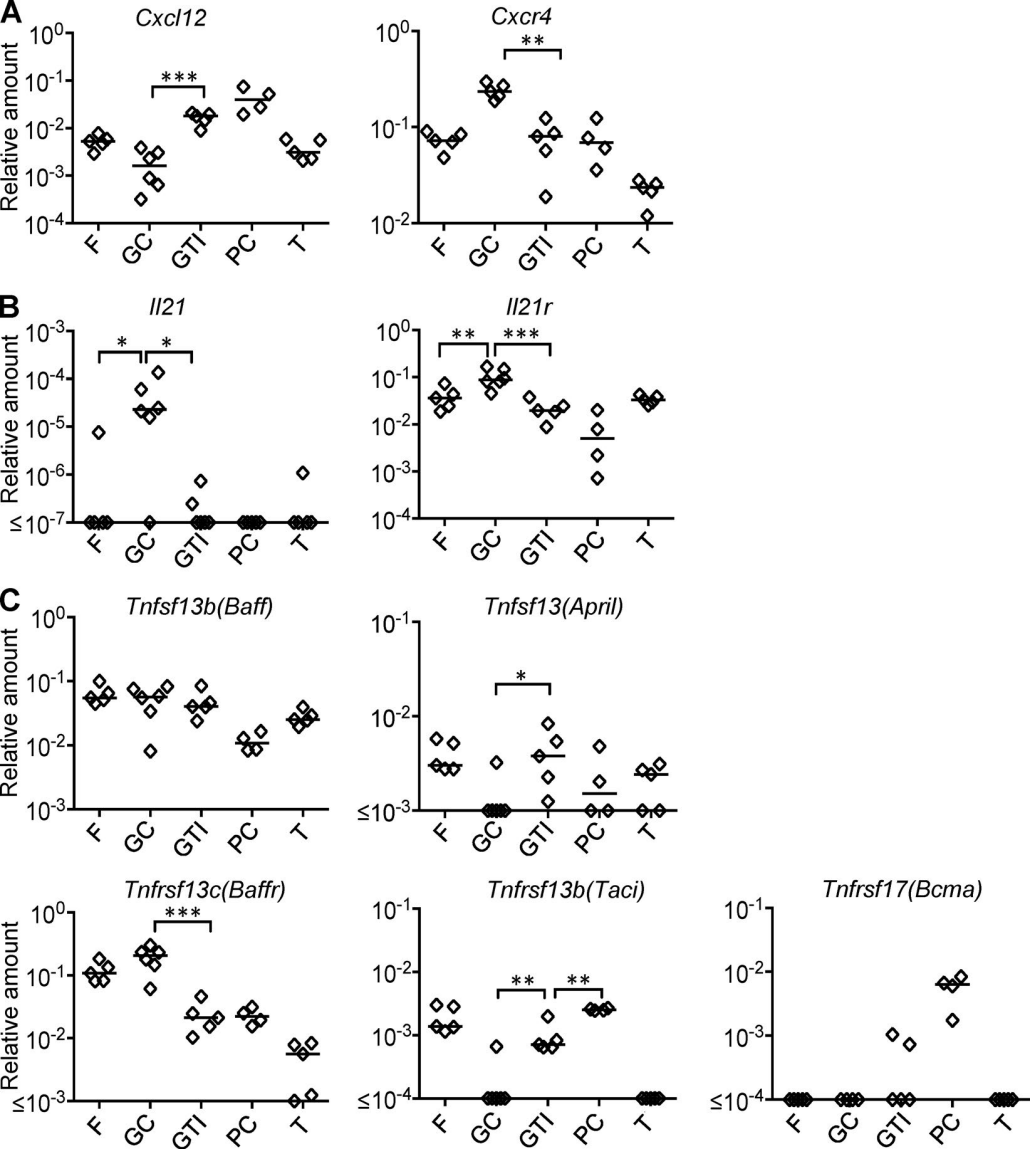
**Gene expression in different microanatomical compartments.** Gene expression was measured by RT-PCR from RNA isolated from laser microdissected parts of spleen sections 5 d after NP-CGG immunization of carrier-primed mice. Areas taken are follicle (F), GC, GTI, extrafollicular plasmablast foci (PC), and central T zone (T). **(A)** Expression of Cxcl12 and Cxcr4 mRNA (**, P = 0.0038; ***, P = 0.0003). **(B)** Expression of *Il21* and *Il21r* mRNA (*, P = 0.0169; **, P = 0.009; *** P = 0.0005). **(C)** Expression of *Baff* and *April* mRNA and their receptors (*, P = 0.02; **, P = 0.0012; ***, P = 0.0002). Expression levels are given as relative amount of mRNA compared with *β2m* mRNA. Each spot represents signal from one area taken from several consecutive spleen sections. Nonpaired two-tailed Student’s *t* test. Two-tailed Mann-Whitney testing used for *Il21, Tnfsf13*, and *Tnfrsf13b* mRNA. Data and statistics are representative of three different spleens.

Detection of chemokines and receptors organizing follicular migration showed, as expected, *Cxcl12* being highly expressed in the GTI and extrafollicular PC areas (Fig. 4 A). The CXCL12 receptor CXCR4, being involved in GC light zone–dark zone translocation, was strongest expressed in GC cells. Although CXCR4 protein was strongly expressed on plasmablasts in the GTI (Fig. 2 I), mRNA expression was not higher in the GTI than in follicular areas, which may reflect posttranscriptional regulation (Al-Souhibani et al., 2014) or dilution of signal by contaminating non-PCs.

Expression of cytokines supporting B cell differentiation (*Il4*, *Il6*, *Il10*, *Il21*, *Tnfsf13* [April], and *Tnfsf13b* [Baff]) was tested. The expression of most cytokines was below detection level, except *Il21, Baff*, and *April* mRNA. *Il21* mRNA was expressed at more than 100× higher levels within microdissected GCs than in other areas (Fig. 4 B). Mean expression of its receptor was also strongest within the GC. *Baff* mRNA was present throughout all B cell areas (Fig. 4 C). Interestingly, *April* mRNA, which is expressed in B cell follicles, was absent from GCs but present in the GTI at similar levels to follicles. *Tnfrsf13c* (*Baffr)* mRNA was strongly expressed in follicles and GCs, but found at 10× lower levels in the GTI. However, mRNA coding for Tnfnrsf13b (TACI), a receptor that binds both BAFF and APRIL, although absent from the GC, was reexpressed in the GTI. Extrafollicular PC foci expressed even higher levels of *Taci* mRNA. Tnfrsf17 (*Bcma)* mRNA, another receptor binding BAFF and APRIL, was detected reliably in extrafollicular PC foci, but there was no significant expression in the GTI. Collectively, these data indicate that B cells developing in the GC receive various differentiation signals, including IL-21 (Shulman et al., 2014). Furthermore, the expression of APRIL, specifically expressed in the GTI, and TACI suggests roles in mediating differentiation and survival in the GTI (Mackay and Schneider, 2008).

### A role for Tfh signals for the differentiation of early GC-derived plasmablasts.

T cell–derived signals have been suggested to have a role for PC differentiation from the GC (Kräutler et al., 2017). IL-21, which is produced by Tfh cells, has been shown to regulate GC development (Linterman et al., 2010; Zotos et al., 2010). IL-21 not only regulates GCs, but also affects early extrafollicular PC appearance independent of GCs (Zotos et al., 2010; Lee et al., 2011). This makes IL-21 a potential regulator of GC-derived plasmablast differentiation.

**Figure 5.**
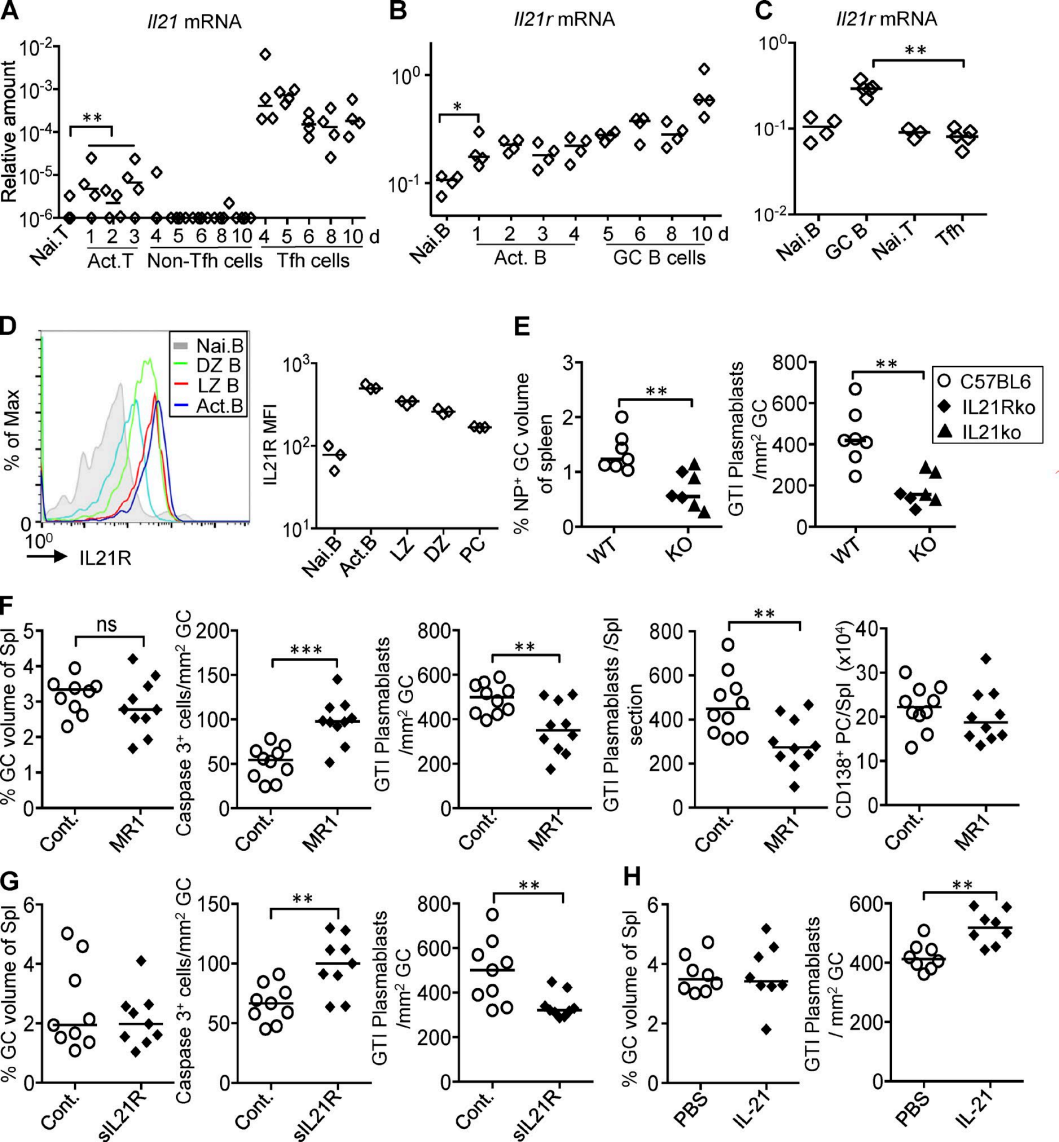
**A role of IL-21 for the differentiation of early GC-derived plasmablasts. (A)** *Il21* mRNA expression in naive CD4^+^ T cells (Nai. T), CD4^+^ CD62L^low^ activated T cells (Act. T), CD4^+^ CXCR5^−^ PD1^−^ non-Tfh cells, and CD4^+^ CXCR5^+^ PD1^+^ Tfh cells at different times after immunization of recipients of eYFP^+^ NP-specific B cells with NP-SRBCs i.v. **, P = 0.0073. **(B)** *Il21r* mRNA expression at different times after immunization in B220^high^ eYFP^−^ naive (Nai. B) or eYFP^+^ activated B cells (Act. B cells) or B220^high^ Fas^high^ eYFP^+^ GC B cells. *, P = 0.028. **(C)** *Il21r* mRNA 5 d after NP-CGG immunization of carrier-primed recipients of eYFP^+^ NP-specific B cells in B220^high^ eYFP^−^ naive B cells, B220^high^ Fas^high^ eYFP^+^ GC B cells, CD4^+^ PD1^−^ non-Tfh cells, or CD4^+^ PD1^+^ Tfh cells. **, P = 0.0079. Two-tailed Mann-Whitney test. **(D)** IL21R expressed on CD86^high^ CXCR4^low^ light zone (LZ) and CD86^low^ CXCR4^high^ dark zone (DZ) B cells measured by FACS at day 5 after SRBC. Each diamond represents one animal. **(E)** GC size and IRF4^+^ plasmablasts in the GTI per GC area assessed from spleen sections of IL21/IL21Rko and wild type (C57BL6) mice 7 d after i.p. injection with NP-KLH in alum. **, P = 0.004. Data merged from two independent experiments. **(F)** Effect of 24 h MR1 treatment 4 d after immunization, showing caspase 3^+^ apoptotic GC cells, GC size, GTI plasmablasts per GC area, and total GTI plasmablasts per spleen section, quantified from immunohistochemically stained spleen sections. Total number of CD138^+^ PCs per spleen measured by flow cytometry. Tissues taken 5 d after immunization with SRBCs i.v. and 24 h after i.v. injection of anti-CD40L or control antibody. **, P = 0.006; ***, P = 0.0002; ns, not significant. **(G)** GC size, GTI plasmablasts per GC area, and Caspase 3^+^ apoptotic GC cells quantified from spleen sections. Tissues taken 5 d after SRBC immunization and 24 h after i.v. injection of IL21R-Fc or control protein. **, P = 0.006. **(H)** GC size and IRF4^hi^ GTI plasmablasts per GC area assessed 5 d after SRBC and 24 h after injection of IL-21 i.v. **, P = 0.0023. Each symbol represents one animal. Data merged from two independent experiments (*n* = 10). Two-tailed unpaired Student’s *t* test.

*Il21* and *Il21r* expression was tested from in vivo–activated T and B cells. To provide larger numbers of antigen-specific B cells, NP-binding B220^+^ cells from quasimonoclonal (QM) enhanced YFP (eYFP) mice were transferred into wild type hosts 1 d before immunization with SRBCs coupled with NP. During the first 3 d after immunization, activated B and T cells were isolated as eYFP^+^ B cells and CD62L^low^ CD4 T cells. During the GC phase, B220^+^ Fas^+^ eYFP^+^ GC B cells and CD4^+^ PD1^+^ CXCR5^+^ Tfh cells were sorted. Despite the role of IL-21 in extrafollicular plasmablast differentiation, activated extrafollicular T helper cells during the first 3 d of the response expressed *Il21* mRNA just above detection level (Fig. 5 A). Tfh cells, isolated from day 4, expressed 100× more *Il21* mRNA than activated Th cells from the extrafollicular phase of the response, with the strongest expression within the first 2 d of the GC response (Fig. 5 A). *Il21r* expression was significantly induced during the extrafollicular phase of B cell activation and increased further once B cells formed GCs (Fig. 5 B). GC B cells isolated at day 5 expressed on average three to four times more *IL21r* mRNA than nonactivated B cells or Tfh cells (Fig. 5 C). Flow cytometry showed increased levels of IL-21R on activated B cells and GC B cells, with slightly higher expression in light zone B cells (Fig. 5 D).

To test the effect of *Il21* deficiency on PC differentiation from the GC, plasmablast numbers in the GTI were quantified 7 d after immunization of *Il21/I21r*-deficient mice with NP coupled to keyhole limpet hemocyanin (NP-KLH). As shown earlier (Zotos et al., 2010), GCs on average were 50% smaller in the absence of IL-21 (Fig. 5 E); the number of GTI plasmablasts, however, was reduced disproportionally more in the absence of IL-21, at 63% reduction in GTI plasmablasts per GC volume (Fig. 5 E). This corresponds to an 83% reduction of total GTI plasmablasts per spleen. Similar results were seen in BALB/c mice deficient in IL-21 receptor (IL21R) after primary immunization with NP-CGG in alum, with a 47% reduction in GC-derived plasmablasts (unpublished data). This points toward IL-21 being a major factor regulating the induction of GC-derived plasmablast differentiation.

To confirm that the emergence of GC-derived plasmablasts is dependent on Tfh–B cell interactions, mice were treated for 24 h with a blocking anti-CD40L antibody. This interval was chosen based on the known kinetics of GC B cell recirculation (Victora et al., 2010) and output (Fig. 3 F). CD40L blocking or control antibody was given i.v. to mice at 4 d after primary SRBC immunization. As expected (Foy et al., 1996), this short blockade led to increased GC B cell apoptosis, but no significant loss of GC volume (Fig. 5 F). Plasmablast output at the GTI was reduced to 70% of control (Fig. 5 F). Importantly, quantification of total CD138^+^ PCs by flow cytometry did not show a reduction within 24 h (Fig. 5 F), indicating that GTI plasmablasts are a population-derived from recent Tfh interactions and, therefore, are sensitive to Tfh signal blockade.

To test the role of IL-21 on plasmablast output more directly, blocking soluble IL-21 receptor was injected, and tissue was analyzed with similar timing as in the CD40L blocking experiment. This led to effects very similar to blockade of CD40 signaling with no significant effect on GC (Fig. 5 G) or extrafollicular PC numbers (not depicted), but effects on B cell apoptosis and plasmablast output through the GTI (Fig. 5 G). Lastly, the effect of IL-21 injection 4 d after SRBC immunization was tested 24 h later. This led to a significant increase of GC-associated plasmablasts without changing GC size (Fig. 5 H). Collectively, these results demonstrate that T cell–B cell interaction and IL-21 are major factors regulating plasmablasts emerging from GCs at the GTI.

### The GTI contains a stromal cell niche that can produce APRIL

**Figure 6.**
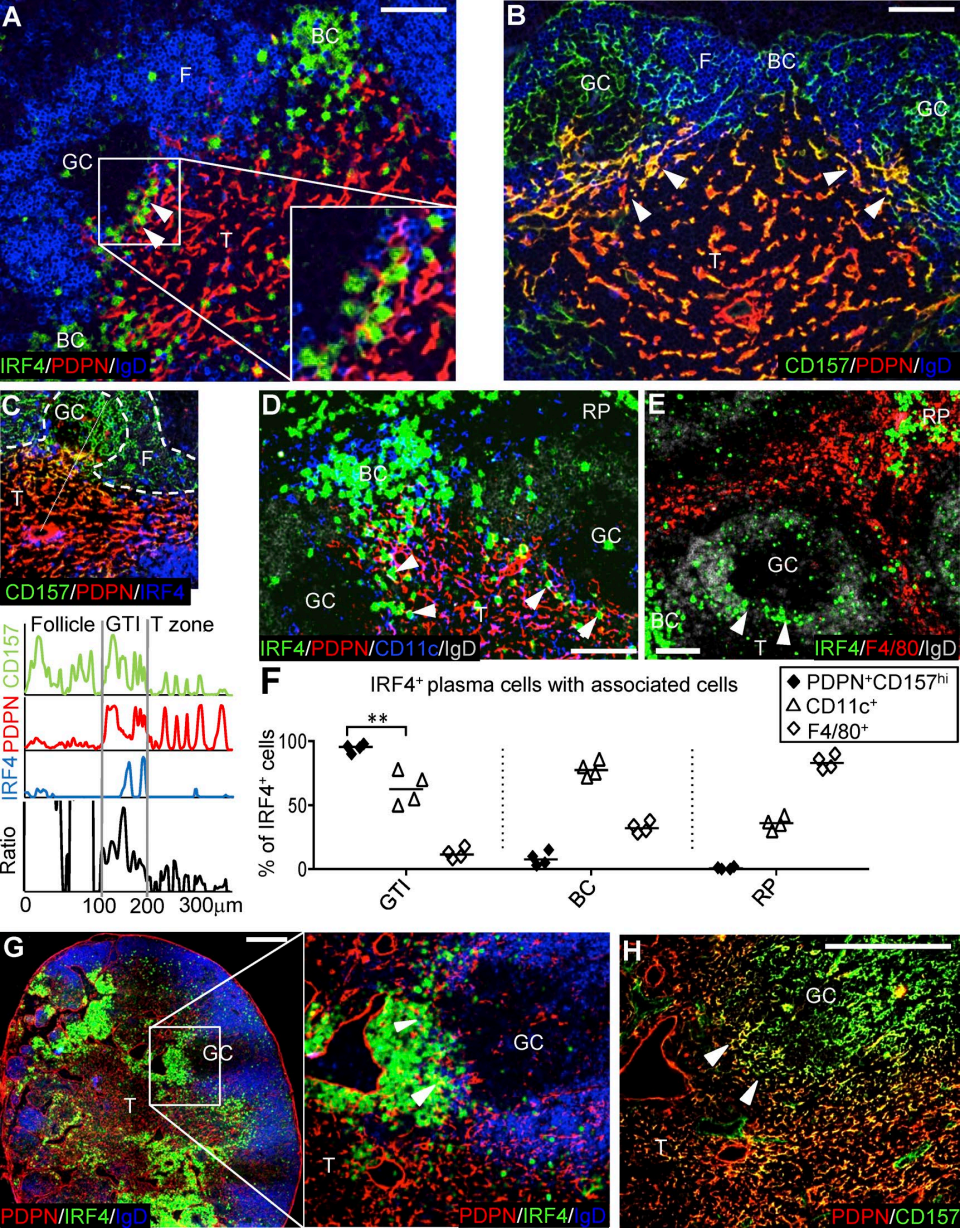
**IRF4^hi^ PCs in close contact with PDPN^+^ CD157^hi^ reticular cells in the GTI of spleen and lymph node. (A)** Triple immunofluorescence of spleen sections 5 d after SRBC immunization stained for IgD on follicular B cells, IRF4 in plasmablasts, and PDPN on T zone stroma. **(B)** Adjacent section stained for IgD, PDPN, and CD157 on follicular stroma and GTIRC. **(C)** Semiquantitative estimate of CD157 staining intensity along a line from the CD157^high^ follicle to the center of the T zone. Ratio: CD157 staining intensity divided by PDPN staining intensity. **(D)** CD11c and PDPN staining to illustrate different stroma associated with IRF4^hi^ PCs in the GTI and red pulp bridging channels. **(E)** F4/80 and IRF4^hi^ PCs in red pulp. Bar, 50 µm. **(F)** Quantification of IRF4hi cells in close contact with PDPN^+^ CD157^+^ reticular cells, CD11c^+^ cells, and F4/80^+^ cells in different areas. Each symbol represents one spleen section. Two-tailed unpaired Student’s *t* test. **, P = 0.0034. **(G)** Large numbers of IRF4^hi^ cells present in the GTI of a popliteal lymph node 8 d after subcutaneous foot immunization with NP-CGG. Bar, 200 µm. Close-up showing IRF4^hi^ cells in contact with PDPN^+^ reticular cells in the GTI (arrowheads). **(H)** Adjacent section stained for PDPN and CD157 shows reticular cells in the GTI coexpressing PDPN and high levels of CD157 (arrowheads). Bar, 50 µm; also applies to magnified panel in G. BC, bridging channel; F, Follicle; RP, red pulp; T, T zone.

Stromal cells have been shown to play a role in directing GC cell migration (Bannard et al., 2013) and to support lymphocyte survival and differentiation (Cremasco et al., 2014; Fasnacht et al., 2014; Zehentmeier et al., 2014). Plasmablasts emerging at the GTI were in intimate contact with podoplanin (PDPN)-positive fibroblastic reticular cells (FRCs; Fig. 6 A). FRCs in the GTI differed from FRCs in the center of the T zone with higher expression of CD157 (Fig. 6 B), usually associated with follicular stroma (Cyster et al., 2000). FRCs within the T zone expressed lower levels of CD157, and this decreased toward the central arteriole (Fig. 6 C). Splenic GTI stroma typically was oriented parallel to the follicle–T zone border, with some extensions penetrating into the GC (Fig. 6 B). PDPN^+^ CD157^+^ cells did not occur in other local niches of PC development. Instead, other cells associated with PC survival were observed in these sites: splenic bridging channels (Fig. 6 D) contained CD11c^high^ dendritic cells (García de Vinuesa et al., 1999), while in the red pulp (Fig. 6 E), F4/80^+^ myeloid cells (Mohr et al., 2009) were found. These cell types were less abundant in the GTI (Fig. 6 F). CD157^high^ PDPN^+^ stroma was also found in the GTI of lymph nodes (Fig. 6, G and H).

**Figure 7.**
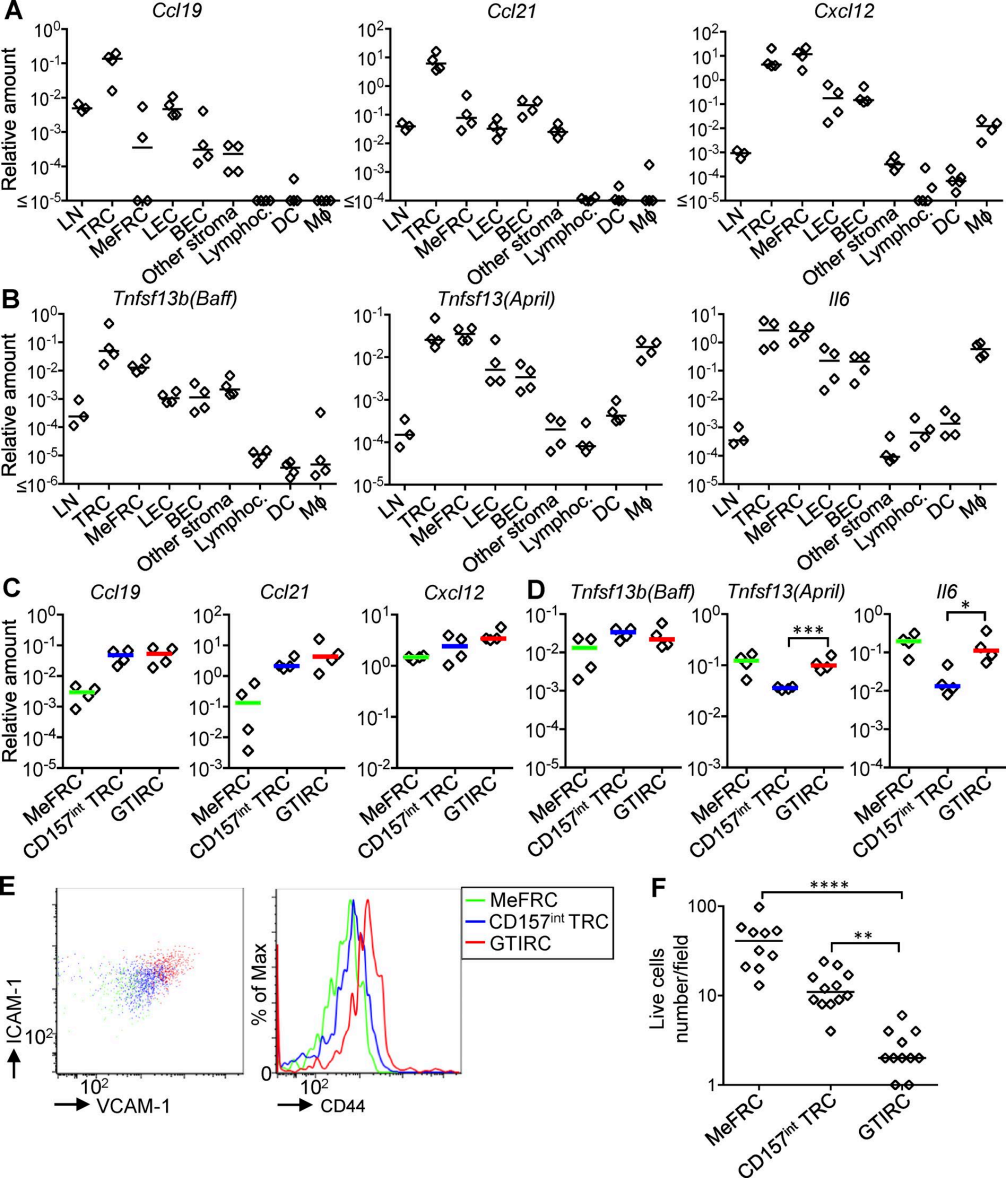
**GTIRC produce cytokines and chemokines supporting GC-derived plasmablasts. (A)** Expression levels of *Ccl19*, *Ccl21*, and *Cxcl12* mRNA in total lymph node cells (LN). CD45^−^ Ter119^−^ EpCAM^−^ stromal cells subdivided into PDPN^+^ CD31^−^ CD157^+^ TRCs, PDPN^+^ CD31^−^ CD157^−^ MeFRC, PDPN^+^ CD31^+^ LECs, PDPN^−^ CD31^+^ BECs, or other stroma not staining for these marker combinations. CD19^+^ CD5^+^ lymphocytes (Lymphoc.), CD11c^+^ MHCII^+^ CD86^+^ DCs, and CD11b^+^ CD11C^−^ macrophages (Mφ) were also sorted. **(B)** Gene expression of *Baff*, *April*, and *Il6*. Each diamond represents pooled popliteal lymph nodes from for mice. **(C)** Chemokine mRNA and **(D)** cytokine mRNA expression after subdivision of T zone PDPN^+^ CD31^−^ CD157^+^ TRC into CD157^high^ GTIRC and CD157^int^ TRC. *, P = 0.01; ***, P = 0.0004. Gating, see Fig. S3. Each diamond represents pooled cells from 12 lymph nodes. All values are relative to the *b2m* mRNA. Data are representative of two independent experiments. Two-tailed unpaired Student’s *t* test. **(E)** Expression of VCAM1, ICAM1, and CD44 on the same stromal cell groups as in C and D. **(F)** Live cells numbers after 48 h culture of isolated stroma cell populations in vitro. **, P = 0.0046; ****, P < 0.0001. Each diamond represents one field; data merged from three independent culture wells. Kruskall-Wallis test comparing nonparametric multiple groups.

The microdissection experiments showed that the GTI is rich in *April* mRNA. To test whether the stromal cells in the GTI are sources of TACI ligands, cells were flow sorted from popliteal lymph nodes 8 d after primary immunization with NP-CGG in alum and *Bordetella pertussis*. CD45^−^, EpCAM^−^, CD31^−^, and PDPN^+^ stroma was separated first from CD31^+^ lymphatic endothelial cells (LECs) and blood endothelial cells (BECs; Link et al., 2007) and then into MadCAM^−^ CD157^−^ medullary FRCs (MeFRCs) and MAdCAM^−^ CD157^+^ T zone reticular cells (TRCs). This TRC fraction should contain the CD157^high^ GTI-associated stroma (Fig. 7 A). Furthermore, lymphocytes, CD11c^+^ dendritic cells (DCs), and CD11b^+^ macrophages were sorted. As expected (Luther et al., 2000), TRC expressed the highest amounts of *Ccl9* and *Ccl21* mRNA (Fig. 7 A). They also expressed high amounts of *Cxcl12* (Fig. 7 A), which may attract CXCR4^+^ plasmablasts emerging from the GC to make contact. TRCs contained at least 9× more *Baff* mRNA than most other stromal populations (Fig. 7 B). Their *April* mRNA levels were only matched by MeFRC and macrophages (Mohr et al., 2009). This shows that stroma in the GTI may well be able to specifically support plasmablast differentiation through TACI signals. Further, TRCs produced high levels of *Il6* mRNA, which is a cytokine that can collaborate with IL-21, inducing PC differentiation (Dienz et al., 2009).

To enrich reticular cells in the GTI (GTIRCs), separating them from inner T zone TRCs, TRCs were subdivided according to CD157 expression into CD157^int^ TRCs and CD157^high^ cells (Fig. S3 A). This confirmed the high expression of *Ccl19*, *Ccl21*, and *Cxcl12* mRNA in GTIRCs, distinguishing them from MeFRC (Fig. 7 C). Although there was no difference in *Baff* mRNA expression between CD157^int^ TRC and GTIRC (Fig. 7 D), *April* and *Il6* were more abundantly expressed in the GTIRC-enriched population, comparable to expression levels found in medullary stroma (Fig. 7 D), known to support PC development (Mohr et al., 2009). Flow cytometry also showed that the GTIRC fraction expressed higher levels of ICAM-1, VCAM-1, and CD44 (Fig. 7 E). Experiments designed to test their function in vitro failed, because, different from CD157^int^ FRC, the CD157^high^ fraction did not survive in isolation (Fig. 7 F and Fig. S3, B and C). These data indicate that stroma in the GTI is different from central T zone TRCs and has the potential to interact with GC-derived plasmablasts via adhesion molecules, chemokines, and cytokines.

### APRIL produced by stroma in the GTI supports plasmablast differentiation.

**Figure 8.**
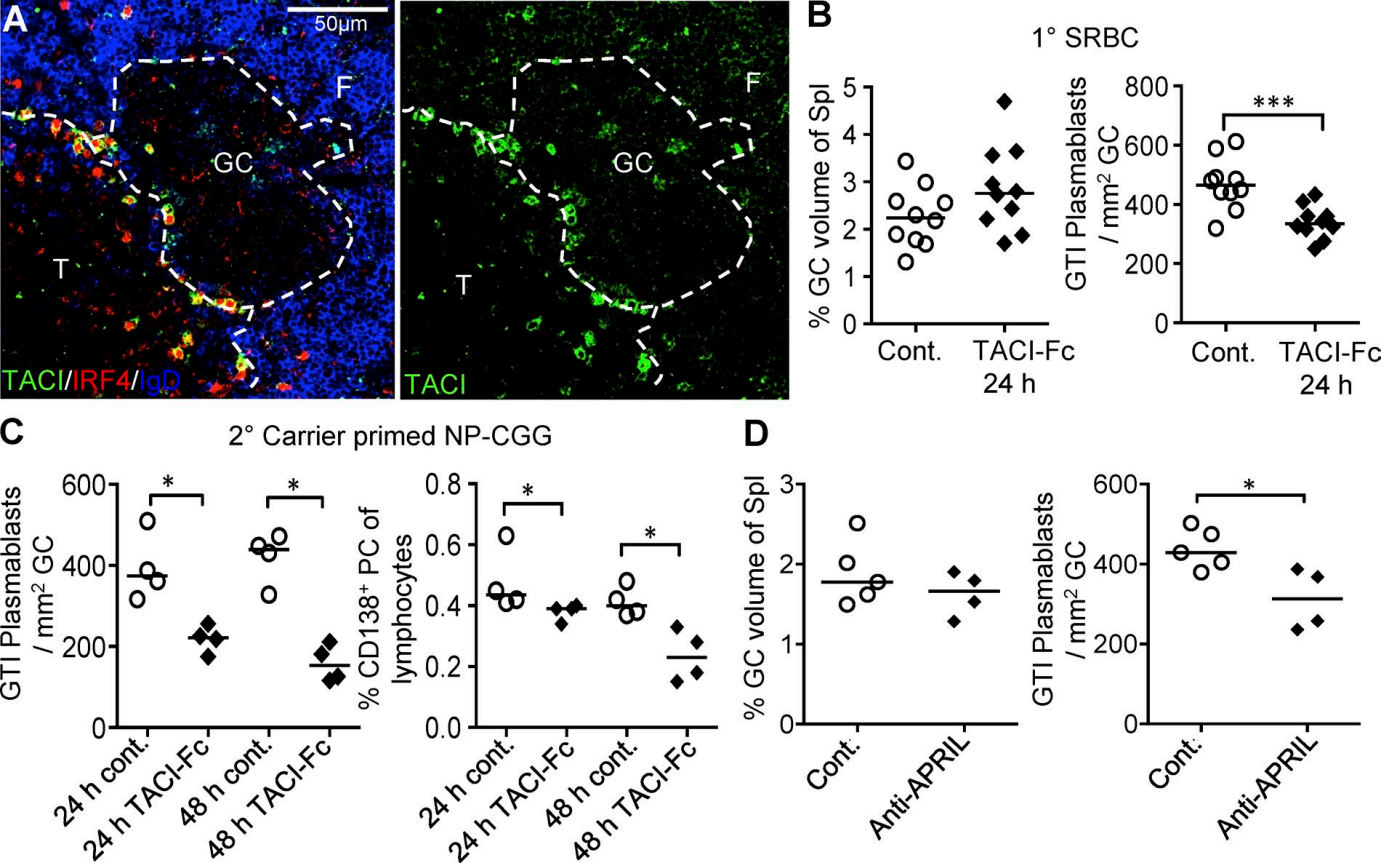
**The effect of blocking TACI ligands on the production of GC-derived plasmablasts. (A)** Triple immunofluorescence histology (left) for TACI, IRF4, and IgD or single channel immunofluorescence for TACI (right) showing low TACI expression in follicles (F) and strong TACI expression in GTI plasmablasts. T, T zone. Dashed lines indicate outline of the T zone and GC. **(B)** The effect of TACI-Fc on the GC size and GTI plasmablasts per GC area assessed in spleen sections. Tissues taken 5 d after immunization with SRBCs i.v. and 24 h after i.v. injection of TACI-Fc fusion protein or control protein mouse Fc from IgG2c. Data from two independent experiments. **(C)** The effect of TACI-Fc on IRF4^hi^ GTI plasmablasts per GC area assessed in spleen sections. Tissues taken 5 d after immunization of carrier-primed mice with NP-CGG i.p. and 24 h or 48 h after i.v. injection of TACI-Fc or control protein. **(D)** The effect of anti-APRIL on IRF4^hi^ GTI plasmablasts per GC area assessed in spleen sections. Each symbol represents one animal. Nonpaired two-tailed Student’s *t* test.*, P = 0.02; ***, P = 0.0009.

The above data indicate that APRIL produced in the GTI supports plasmablast output. Immunohistology of spleen sections taken 5 d after SRBC immunization confirm the pattern of TACI expression seen by microdissection: TACI is expressed at low levels in follicular B cells, absent on GC B cells, and is strongly expressed on plasmablasts emerging in the GTI, as well as on extrafollicular PCs in the bridging channels and the red pulp (Fig. 8 A). To test whether TACI signaling regulates emergence of plasmablasts from GCs, mice were injected with TACI-Fc fusion protein 4 d after primary immunization with SRBCs. TACI-Fc is a soluble decoy for BAFF and APRIL, and hence may abrogate ligand-mediated signaling through all BAFF and/or APRIL receptors (Bossen et al., 2008). Although within 24 h this did not lead to significant effects on GC size, there was a 30% reduction of GTI plasmablast numbers (Fig. 6 B). Parallel experiments in mice undergoing carrier-primed responses to NP-CGG that were treated with TACI-Ig for 24 or 48 h before analysis on day 5 showed that GTI plasmablast numbers were reduced by 42 and 62%, respectively (Fig. 6 C), whereas there was no reduction in GC size (not depicted). TACI signals are essential for PC survival, which should lead to loss of PCs in red pulp and bridging channels. This, however, was only obvious after 48 h TACI-Ig blockade (Fig. 8 C). To test whether APRIL is the TACI ligand supporting emergence of PCs in the GTI, the experiment was repeated using anti-APRIL blocking antibody, which again led to a significant reduction of plasmablasts in the GTI within 24 h (Fig. 8 D), supporting the conclusion that GTIRC-produced APRIL supports plasmablast differentiation in the GTI.

## Discussion

We show here that plasmablasts emerge locally from the earliest stages of the GC response into a specialized microenvironment, which is the border between GC dark zone and T zone, or GTI. From here they may migrate to other local survival niches such as the splenic extrafollicular foci or the lymph node medulla or to the bone marrow. We show that the process of plasmablast differentiation starts very early during GC development and that IL-21 and TACI ligands regulate this process. While IL-21 is well known to be produced by Tfh cells, APRIL is produced by a new CD157^high^ PDPN^+^ stromal cell population located in the GTI. These GTIRCs are in close contact with plasmablasts emerging at this site. GTIRCs also produce mRNA coding for CXCL12, BAFF, and IL-6, which may attract and then support differentiating plasmablasts.

During their several weeks of existence, GCs mature. Not only does the affinity of GC B cells and antibody increase over time, GCs also seem to vary their main types of output cells: a recent study shows preferential output of affinity-matured long-lived PCs that persist in bone marrow at late stages of the GC response, whereas younger GCs preferentially produce memory B cells (Weisel et al., 2016). We have not tested whether PCs seen in the GTI during the earliest stages of the GC reaction home to bone marrow or not. The findings by Weisel et al. (2016) suggest plasmablasts in the GTI may differentiate only locally and do not migrate to bone marrow, or it is also possible that GC-derived PCs first replace PCs in local niches and only at later stages start to appear in the bone marrow. Recent studies have shown that PC differentiation in the GC is induced in higher-affinity B cells (Kräutler et al., 2017), whereas memory B cell output is delayed and of lower quality (Goenka et al., 2014b; Suan et al., 2017). The higher-affinity antibody produced at an early stage of the GC response may not only provide rapid defense against pathogens, but also function by regulating the GC itself. Antibodies reentering GCs can restrict accessibility of the antigen on follicular dendritic cells and raise the GC B cell selection threshold through antibody feedback (Zhang et al., 2013). It is not known whether this antibody reenters GCs by active transport of diffusion. Antibody-producing cells located in the GTI are placed ideally to provide such antibodies locally.

A strong drive toward plasmablast differentiation at the earliest stages of the GC response may reflect the easy access to antigen held on follicular dendritic cells, which at this stage is still complexed by low-affinity antibody derived from the early extrafollicular PC response (Sze et al., 2000). Indeed, earlier experiments show that PC output from GCs is reduced once GC B cells incur higher-stringency antibody feedback (Zhang et al., 2013).

GC B cell selection depends on antigen access and antigen presentation–dependent Tfh cell signals. Although PC output from the GC is skewed toward higher affinity (Chan and Brink, 2012), B cell receptor signaling seems to have a minor role on GC B cell selection (Khalil et al., 2012). T cell interactions are a major regulator of GC B cell differentiation (Victora and Nussenzweig, 2012), and these interactions are limited by the efficiency with which B cells are able to take up antigen and present this to T cells. Tfh cell signals induce GC B cell recirculation into the dark zone for further proliferation and immunoglobulin gene hypermutation (Victora et al., 2010; Gitlin et al., 2015; Liu et al., 2015). Tfh cells produce IL-21 and IL-4 when interacting with B cells (Shulman et al., 2014).

Several functions are attributed to IL-21. IL-21 shapes Tfh cell differentiation, influencing their capacity to produce IL-4 and IL-21 (McGuire et al., 2015). IL-21 may regulate extrafollicular PC generation during initial B cell activation, but also GC development (Linterman et al., 2010; Zotos et al., 2010). In naive B cells IL-21 can induce GC differentiation and inhibit TACI expression (Goenka et al., 2014a), but it can also cooperate with IL-4 to induce Blimp1, leading to PC differentiation (Ozaki et al., 2004). Therefore, the main function of IL-21 seems to be more a general trigger of B cell differentiation, rather than being instructive for differentiation toward a specific direction (Ozaki et al., 2004). Other factors, e.g., IL-4, may be able to provide additional instructive co-stimuli. The rapid effect of IL-21 on plasmablast differentiation shown here makes it likely that at least part of the action of IL-21 is on B cells directly. Therefore, the reduced affinity maturation seen in IL-21–deficient animals (Zotos et al., 2010) may at least partly be a result of a direct reduction of PC output from the GC. Other models are possible: increased antigen presentation by GC B cells to Tfh cells not only increases recirculation to the GC dark zone, but also PC generation (Victora et al., 2012). Once arrived in the dark zone, asymmetric cell division may drive some cells into PC output (Meyer-Hermann et al., 2012; Thaunat et al., 2012), or contact with stroma at the edge of the GC may signal PC differentiation.

PC differentiation and survival happens in niches of secondary lymphoid tissues (Sze et al., 2000) and bone marrow (Manz et al., 2002) that provide homing signals and survival factors (Chu et al., 2011; Chu and Berek, 2013). Higher-affinity PCs continuously replace the initially formed low-affinity PCs in these compartments (Smith et al., 1997). The composition of these compartments is probably homeostatic in nature, i.e., simply dependent on influx, because of the emergence of new cells, and death, because of limited niche capacity, rather than PC affinity or developmental origin (Sze et al., 2000; Manz et al., 2002; Mohr et al., 2009). Early output of plasmablasts from GC described here may contribute to the replacement of PCs in local niches from a very early stage (Sze et al., 2000).

Stroma in the GTI represents a new niche supporting differentiation of GC-derived PCs. CD157^high^ PDPN^+^ GTI stroma is rich in CXCL12 and CCL19/21. GTIRC may be related to CXCL12-expressing reticular cells (CRCs) located in the dark zone that have been shown to also express CCL19 (Bannard et al., 2013; Rodda et al., 2015). However, CRCs characteristically form long protrusions into the GC dark zone (Rodda et al., 2015), whereas GTIRCs are typically located outside the GC and in the spleen form strands parallel to the border between the GC and T zone. Cells committed to plasmablast differentiation may be attracted by chemokines produced by GTIRC, and plasmablasts in the GTI express high levels of CXCR4 and also CCR7. Plasmablasts in the GTI express TACI, which is not expressed inside the GC (Goenka et al., 2014a). Although BAFF has been shown to regulate affinity-dependent selection in the GC (Goenka et al., 2014a), stroma in the GTI is the initial microenvironment that provides APRIL. TACI ligation by APRIL inhibits B cell proliferation and can induce PC differentiation (Mackay and Schneider, 2008), whereas BAFF present in the GC seems to have exactly the opposite effect (Goenka et al., 2014a). Additionally, GTIRC produce *Il6* mRNA, which collaborates with IL-21 inducing B cells to differentiate into PC (Dienz et al., 2009). APRIL and IL-6 are also produced by downstream stromal niches where PCs differentiate after emerging through the GTI, e.g., macrophages in the lymph node medulla (Mohr et al., 2009).

A simple model for the regulation of PC output summarizing the data presented here would be that B cells successfully selected by Tfh cells (dependent on how well they were able to access and present antigens) and being exposed to Tfh-derived IL-21 plus possible cofactors such as IL-4 (Shulman et al., 2014) will get stimulated to recirculate to the dark zone. There they will proliferate, and some will differentiate into PCs. Whether this is directional differentiation caused by specific instructive signals, asymmetric cell division (Meyer-Hermann et al., 2012; Thaunat et al., 2012), or simply a result of cells getting into contact with chemokine-expressing GTIRCs producing APRIL and IL-6 remains to be seen. In any case, the local environment in the GTI will support these cells to undergo further replication and differentiation, before moving toward longer term survival niches and differentiating into mature nonproliferating antibody–secreting PCs.

## Materials and methods

### Mice and immunizations

6- to 12-wk-old sex-matched C57BL/6 mice were obtained from Harlan Laboratories and kept in specified pathogen-free conditions. For intravital microscopy, Igh^tm2Cgn^ (B1-8i^+/+^) mice (gift from K. Rajewsky, Max Delbrück Center for Molecular Medicine, Berlin, Germany) produce Ig heavy chains that are NP-specific when combined with λ light chains. These were crossed onto κ light chain Igk^tm1Dhu^ (Jκ^−/−^) mice and backcrossed onto C57BL/6 for at least nine generations (Hauser et al., 2007). They were further crossed onto Prdm1^tm1Nutt^ mice, expressing a GFP reporter in the Prdm1 locus (Kallies et al., 2004), and onto Tg(CAG-mKO2/CDT1)596Amiy mice (Sakaue-Sawano et al., 2008), which express a short-lived version of Kusabira-Orange (mKO2) during the G1 phase of the cell cycle. These mice are labeled following B18i/k^−/−^/Prdm1^GFP^/Cdt1^mKO2^ mice. For all other adoptive transfer experiments, mice were used that were homozygous NP-specific Ig heavy chain–variable region from Igh-J^tm1(VDJ-17.2.25)Wabl^ (Cascalho et al., 1996), Jk^−/−^ and contain a constitutively expressed eYFP derived from Gt(ROSA)26Sor^tm1.1(EYFP)Cos^ (QM eYFP mice; Srinivas et al., 2001). IL-21 (Il21^tm1Lex^)- and IL-21R (Il21r^tm1Wjl^)-deficient mice are described in (Zotos et al., 2010). *Aicda* ^CreERT2^ mice (gift from C.-A. Reynaud, Institute Necker, Paris, France; Dogan et al., 2009), were crossed with ROSA^mT/mG^ mice (007576; Jackson Laboratory), which contain a Cre-inducible membrane-tagged version of eGFP (Muzumdar et al., 2007). R26R*-*confetti/Cre^ERT2^ (Snippert et al., 2010) spleen sections were from Thomas Winkler (Friedrich-Alexander-University Erlangen-Nuremberg, Erlangen, Germany).

Animal studies were performed with approval of the Birmingham Ethical Review Subcommittee and under a UK Home Office project license. Intravital imaging experiments were conducted according to German animal protection laws and approved by the appropriate governmental authority (Landesamt für Gesundheit und Soziales) in Berlin.

For primary immunizations, mice were injected i.p. with 50 µg alum-precipitated NP-coupled to CGG at a molar ratio of 18:1 plus 10^7^ chemically killed *B. pertussis* (Lee Labs; Becton Dickinson) (Sze et al., 2000) or 100 µg alum-precipitated NP-KLH at a ratio of 17:1 i.p. (Zotos et al., 2010), i.v. with 2 × 10^8^ SRBCs (TCS Biosciences), or freshly prepared NP-haptenated SRBCs (NP-SRBCs) in PBS. Carrier-primed responses were induced by injecting 50 µg soluble NP_18_-CGG i.p. into mice that were primed with CGG 4 wk earlier (Sze et al., 2000). Recombination of the mT/mG allele in *Aicda* ^CreERT2^ mice was induced by a single gavage of 6 mg tamoxifen (Sigma) dissolved in corn oil at 20 mg/ml 4 d after SRBC immunization, and tissues were analyzed 24 or 48 h later. The R26R-Confetti/Cre^ERT2^ mice were induced three times by gavage of 4 mg tamoxifen and on the fourth day immunized with SRBCs i.v. GCs were analyzed 5 d after immunization.

### Immunohistology

Spleen sections were prepared and double-stained as described previously (Marshall et al., 2011). The following additional antibodies were used: goat anti–mouse IRF4 (M-17; Santa Cruz Biotech) and rabbit anti–mouse active caspase 3 (C92-605; BD Biosciences), followed by biotinylated donkey anti–sheep or swine anti–rabbit antiserum (Dako) and StreptABComplex/AP as described (Sze et al., 2000). In the final step, color was developed under visual inspection using FastBlue and 3,3′-Diaminobenzidine (Sigma-Aldrich).

For fluorescence staining, IgD-FITC, B220-FITC (RA3-6B2), CD21-FITC (7G6), BCL6-Alexa488 (K112-91), CD138 (281-2), and CXCR4 (2B11) were from BD Bioscience. Blimp1 (6D3; Santa Cruz Biotech), IgG (MCA424; Serotech), CD38-Alexa647 (90; BioLegend), biotinylated peanut agglutinin (Vector), Ki-67 (Abcam), and IgD-Alexa647 (11-26; eBioscience) were used. Secondary antibodies were FITC-conjugated donkey anti–rat or donkey anti–rabbit, Cy3 or Cy5 conjugated donkey anti–sheep, and Cy3-conjugated goat anti–hamster (Jackson ImmunoResearch). The slides were mounted in antifade mounting medium (Prolong Gold; Invitrogen). Images were taken on fluorescence microscope (DM6000; Leica). Image data were analyzed using Fiji (Schindelin et al., 2012) or point counting using a microscope with an eyepiece containing a counting graticule (Weibel, 1963). Plasmablasts at the GTI were quantified on IRF4/IgD double-stained tissue sections by counting all IRF4^high^ cells in a 40-µm wide strip along the GTI (Fig. S4). Cell numbers were divided by the area of IgD^−^ GCs on the same tissue section.

Confetti spleen sections were stained with goat anti–mouse IRF4, followed donkey anti–goat Alexa Fluor 594 (Invitrogen). Some sections were counterstained with IgD APC (BD Bioscience). A confocal microscope (LSM880; Zeiss) was used to separate six colors with excitation/detection wavelengths: mCerulean (CFP) 405/420 ± 5 nm, hrGFPII (GFP) 488/503 ± 13 nm, mYFP (YFP) 514/530 ± 13 nm, tdimer2(12) (dsRFP) 561/583 ± 13 nm, Alexa Fluor 594 594/630 ± 10, and APC 633/692 ± 43 nm.

### Laser capture microdissection for semiquantitative RT-PCR (qRT-PCR)

qRT-PCR gene expression analysis from laser capture microdissected tissue was done from snap-frozen acetone-fixed spleen sections, taken 5 d after i.p. NP-CGG immunization of carrier-primed C57BL/6 mice. Between 10 and 20 8-µm thick serial sections were collected on photoactivated localization microscopy membrane slides (NF; Zeiss) hydrated in 100, 70, and 50% ethanol and stained for 3 min with 1% wt/vol cresyl violet (Sigma-Aldrich). Slides were then dehydrated by quick washes in 50, 70, and 100% ethanol and air-dried. Laser capture microdissection was performed using a Microbeam HT microscope (Zeiss). To identify GCs and GTI unequivocally, four sections taken from the series were stained immunoenzymatically for IgD and IRF4 (Zhang et al., 2016). A photograph of each section was printed and used as a reference to identify GC (IgD^−^ area within IgD^+^ follicles), follicles (IgD^+^), T zone (IgD^−^ areas surrounded by follicles with central arteriole), the GTI (between GC and T zone containing IRF4^+^ cells), and plasmablast/PC-rich extrafollicular foci (IRF4^+^ between T zone and red pulp; Fig. S2). Membrane-only areas were selected as a negative control. Serial microdissected areas were catapulted into RNeasy buffer (Qiagen) in the nuclease-free microtiter plate lids. RNA was isolated immediately using RNeasy Micro kit (Qiagen). cDNA was stored at −20°C, and qRT-PCR gene expression analysis was done as described (Zhang et al., 2013). Sequence of primers and probes are listed in Table S1.

### Two-photon laser-scanning microscopy

B cells from B18i/k^−/−^/Prdm1^GFP^/Cdt1^mKO2^ spleens were isolated using EasySep B cell untouched isolation kit (StemCell Technologies). 3 × 10^6^ B cells were injected i.v. into C57BL6/J recipients 1 d before immunization with 10 µg NP-CGG emulsified in complete Freund’s adjuvant into the right foot. To label follicular dendritic cells, 10 µg Alexa Fluor 633–labeled CD21/35 Fab fragments were injected into the same foot 12–24 h before imaging. To identify B cell follicles, naive B cells from C57BL6 spleens labeled with 2.5 µM Hoechst 33342 (Invitrogen) were injected i.v.

Mice were anesthetized by i.p. injection of 0.1 mg ketamine and 0.01 mg xylazine (Rompun; Bayer Healthcare) per gram body weight. If necessary, anesthesia was topped up by further i.m. injection of anesthetic. Surgical preparation of the mouse popliteal lymph node was performed as already published (Mempel et al., 2004). In vivo imaging was performed with a two-photon laser-scanning system (LaVision BioTec) equipped with an optical parametric oscillator (APE). The system was pumped with a femtosecond-pulsed titanium-sapphire laser, and excitation wavelength was 930 nm. An objective lens for deep tissue imaging (20× dipping lens, NA 0.95, WD 2 mm; Olympus) was used. XYZ-stacks were collected within a scan field of 500 × 500 µm at 512 × 512-pixel resolution and a Z-plane distance of 3 µm. The fluorescence signal was detected with photomultiplier tubes with the following interference filters: 460 ± 30 nm, 525 ± 25 nm, 593 ± 20 nm, and 655 ± 20 nm.

### Cell sorting for qRT-PCR

For sorting of activated B and T cells, 10^5^ QM eYFP cells were adoptively transferred into C57BL6 hosts 1 d before immunization with NP-SRBCs i.v. Splenocytes were stained using Hoechst 33258 (Sigma-Aldrich), B220 APCCy7 (RA3-6B2, BioLegend), anti–Fas-PECy7 (Jo2), CD4-APC (RM4-5), CD62L-PE (MEL-14; BD Biosciences), PD-1-PE (J43, eBioscience), CXCR5-biotin, and Streptavidin-PerCPCy5.5 (BD Biosciences). Cell populations were sorted in a high-speed cell sorter (MoFlo; Beckman-Coulter). Until 4 d after immunization, activated B cells were sorted as B220^hi^ eYFP^+^, activated T cells as CD4^+^ CD62L^low^, and nonactivated T cells as CD4+CD62L^+^. From day 5, GC B cells were sorted as B220^hi^ eYFP^+^ Fas^hi^, Tfh cells as CD4^+^ PD-1^+^ CXCR5^+^, and non-Tfh cells as CD4^+^ PD-1^−^ CXCR5^−^.

Lymph node stromal cell populations were done with variations as described (Link et al., 2007). In brief, popliteal lymph nodes dissected into small pieces and digested by shaking for 45 min at 35°C in 1 ml RPMI 1640 medium containing 10% FCS, 1% penicillin-streptomycin, 0.1 mg DNaseI (Sigma-Aldrich), and 2.5 mg Collagenase D (Roche). Not fully digested tissue was incubated for another 20 min with 1 ml of fresh digestion buffer. Enzymatic digestion was completed by adding 15 µl 0.5 M EDTA left on ice for 5–10 min. Cells were filtered and washed with PBS (0.5% FCS and 2 mM EDTA). To enrich the stromal cell fraction, hematopoietic cells were depleted by incubating the cell suspension with MACS anti-CD45 microbeads and passing over a MACS LS column (Miltenyi). The enriched cells were incubated for 20 min at 4°C in PBS containing 0.5% BSA and 2 mM EDTA with the following fluorescently labeled antibodies: CD45-PerCPCy5.5 (C363-16A), Ter119-PerCPCy5.5 (Ter119), EpCAM-PerCP-Cy5.5 (G8.8), CD157-APC (BP-3), VCAM-1 PECy7 (429), CD44 Alexa Fluor 700 (IM7), Str BV605 (BioLegend), PDPN-PE (eBio8.1.1), CD31-FITC (390), MAdCAM-1-biotin (MECA-367), ICAM-1 pacific blue (YN1/1.7.74; eBioscience), and Str-PECy7 (BD Biosciences).

CD45^−^ Ter119^−^ EpCAM^−^ were gated as stroma. From this population, further subpopulations were sorted as follows: TRCs as CD31^−^ PDPN^+^ CD157^+^ and MAdCAM^−^ (Mempel et al., 2004), GTIRCs as MAdCAM^−^ CD31^−^ PDPN^+^ CD157^hi^, MeFRC as PDPN^+^ CD31^−^ CD157^−^, LECs as PDPN^+^ CD31^+^, BECs as PDPN^−^ CD31^+^, and other stroma as cells not staining for these marker combinations. Further populations sorted were: other lymphocytes as CD19^+^ CD5^+^, DCs as CD11c^+^ MHCII^+^ CD86^+^ and macrophages as CD11b^+^ CD11c^−^.

Stromal cells were sorted by using low-pressure in a MoFlo Astrios (Beckman Coulter), cDNA preparation was as described as before (Link et al., 2007). Real-time PCR from cDNA (qRT-PCR) was done in multiplex with β2-microglobulin (β2m) and gene expression related to β2m expression levels. Primers and probes are listed in Table S1.

### In vivo treatment with antibodies, fusion proteins, or cytokines

250 µg of hamster anti-mouse CD40L blocking antibody (MR1; gift from N. Jones, University of Birmingham, Birmingham, England, UK), control antibody hamster IgG (Jackson ImmunoResearch), soluble IL-21R fusion protein, or mock protein (mouse IgG2a, gift from L. Walker, University College London, London, England, UK) were injected in 150 ml PBS (i.v.); 1 µg of IL-21 cytokine (Peprotech) was injected i.v.

TACI-Fc is a fusion protein comprising the extracellular portion of TACI and the FC portion of mouse IgG2c, which had been mutated to eliminate complement fixating capacity and binding to Fc receptors. Mouse Fc from IgG2c (mFc) was used as control protein. 100 µg of TACI fusion protein (TACI-Fc) or mFc as control or 90 µg of anti-APRIL (Apry-1-1; AdipoGen), or isotype control mouse IgG2b (Jackson ImmunoResearch) were injected i.v.

Antibody, cytokines or soluble receptors were injected 4 d after SRBC immunization. Tissues were harvested 24 h later. TACI-Fc and control Fc were injected 48 h or 24 h before endpoint at 5 d after NP-CGG immunization of carrier-primed mice.

### Statistical analysis

All statistical analysis was performed on Prism 6 using nonpaired two-tailed Student’s *t* test from log transformed data or two-sided Wilcoxon Mann-Whitney U Test where indicated. Statistics were done by including data from all independent replicates. P values are indicated throughout with * for P < 0.05, ** for P < 0.01, *** for P < 0.001, and **** for P < 0.0001.

### Online supplemental material

Fig. S1 shows the frequency and principal component analysis of florescent protein cells in GCs and adjacent GTIs. Fig.S2 shows the validation of microdissection and qRT-PCR method for different areas of immunized spleen. Fig. S3 shows the isolation of fibroblastic reticular cell subpopulations. Fig. S4 shows the manual stereological analysis of tissue sections and its validation by digital image analysis.

## Supplementary Material

Supplemental Materials (PDF)
