# Supplementary material for: Plasma cell output from germinal centers is regulated by signals from Tfh and stromal cells
Source: J Exp Med. 2018 Apr 2;215(4):1227–43. doi: 10.1084/jem.20160832 (PMC5881458; doi:10.1084/jem.20160832)
Supplement: Supplemental Materials (PDF) [file JEM_20160832_sm.pdf]

Supplemental material

Zhang et al., https://doi.org/10.1084/jem.20160832

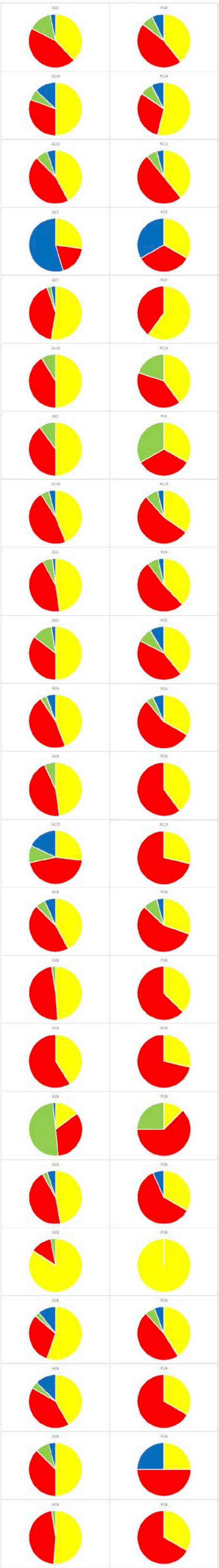

PC1	# GC	# GTI PC	PC1
0.033	311	28	0.028
-0.158	62	13	-0.184
0.002	328	46	0.054
-0.089	11	3	-0.027
-0.101	38	5	-0.171
-0.083	34	5	-0.009
-0.090	20	3	0.006
-0.001	121	26	0.114
-0.045	162	29	0.075
-0.120	82	23	0.011
-0.010	301	45	0.132
-0.039	44	5	0.110
0.109	67	7	0.271
-0.001	223	23	0.163
-0.028	45	8	0.146
0.098	44	7	0.271
0.153	60	8	0.349
-0.041	98	15	0.158
-0.530	70	3	-0.735
-0.198	168	17	0.018
-0.025	24	3	0.204
-0.109	72	4	0.148
-0.061	43	3	0.204

Figure S1. **Frequency of CFP-, GFP-, YFP-, and RFP-positive cells in 23 GCs and adjacent GTIs.** PC1 is the principle component 1 of a principle component analysis of the four color frequencies using Clustvis (http://biit.cs.ut.ee/clustvis/, accessed December 11, 2017). #GC and #GTI PC are the number of IRF4⁺ XFP⁺ cells detected in each zone that went into the analysis.

JEM

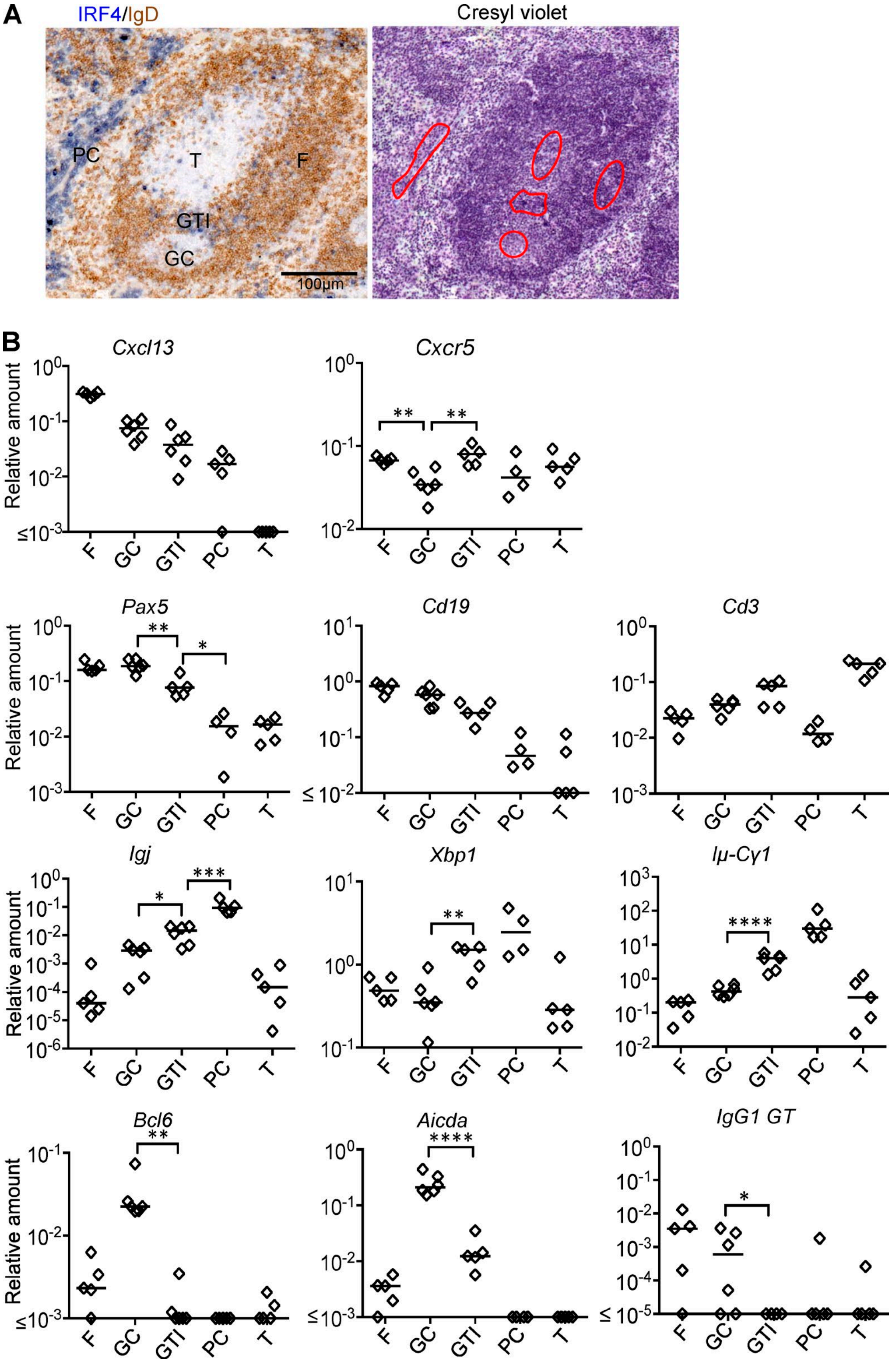

Figure S2. **Validation of microdissection and qRT-PCR method for different areas of immunized spleen. (A)** Photomicrographs of two proximate spleen sections 5 d after NP-CGG immunization of carrier-primed mice. Left, immunohistochemical staining for IgD and IRF4 identifies B cell follicle (F), GC, GTI, PC areas in red pulp (PC), and T zone (T). Right, cresyl violet staining revealing intense staining of the B cell follicle and GTI and weaker staining of the GC, T zone, and red pulp. Red lines indicate examples of areas microdissected for qRT-PCR analysis. **(B)** Relative expression levels of mRNA coding for Cxcl13, Cxcr5, *Pax5, CD3* and *CD19, IgJ, Xbp1* mRNA and *Iμ-Cγ1 hybrid transcript, Bcl6, Aicda,* and *IgG1 germline transcript (IgG1 GT)* from follicle, GC, GTI, PCs in red pulp, and T zone. Each spot represents signal from one specific area taken from several consecutive sections. All values are relative to expression of β2m mRNA. Data and statistics are representative of three different spleens. Nonpaired two-tailed Student's *t* test; *, P = 0.03; **, P = 0.0052; ***, P < 0.0002; ****, P < 0.00001. Two sided Mann-Whitney test for Bcl6, IgG1 GT; *, P = 0.04; **, P = 0.002.

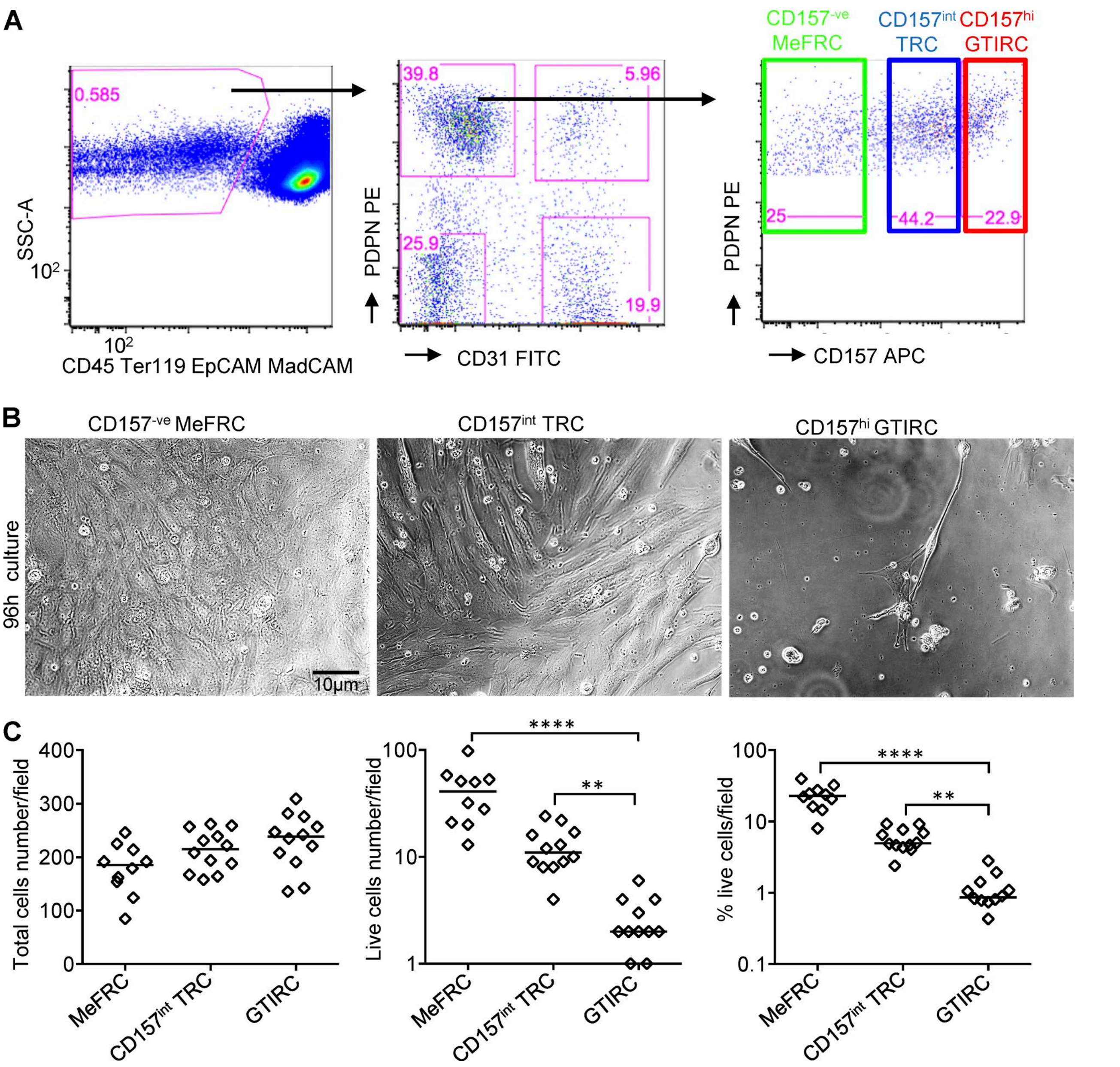

Figure S3. **Isolation of fibroblastic reticular cell subpopulations. (A)** Sorting protocol. Lymph nodes were harvested 8 d after s.c. NP-CGG in alum foot immunization. $CD45^-$ $Ter119^-$ $EpCAM^-$ $MadCAM^-$ stromal cells were subdivided into $PDPN^+$ $CD31^-$ TRCs, $PDPN^+$ $CD31^+$ LECs and $PDPN^-$ $CD31^+$ as BECs. Right, subdivision of TRCs into $CD157^{high}$ GTIRC, $CD157^{int}$ TRC, and $CD157^{-ve}$ MeFRC. **(B)** Representative microphotographs from $CD157^{-ve}$ MeFRCs, $CD157^{int}$ TRCs, and $CD157^{high}$ GTIRCs after 96 h culture. Reticular cells were sorted into three populations according to the protocol, 10,000 freshly sorted cells plated into αMEM with 10% FBS and 1% pen/strep, and incubated at 37°C/10% CO_2. **(C)** Cells were counted using a light microscope (20×) after 48 h, 1 field was counted. Cells were scored as attached and elongated (alive) or round, often with membrane blebbing evident (Fletcher et al., 2011). Each diamond represents one field. Data merged from three independent culture wells. Kruskall-Wallis test comparing nonparametric multiple groups. **, P = 0.0046; ****, P < 0.0001.

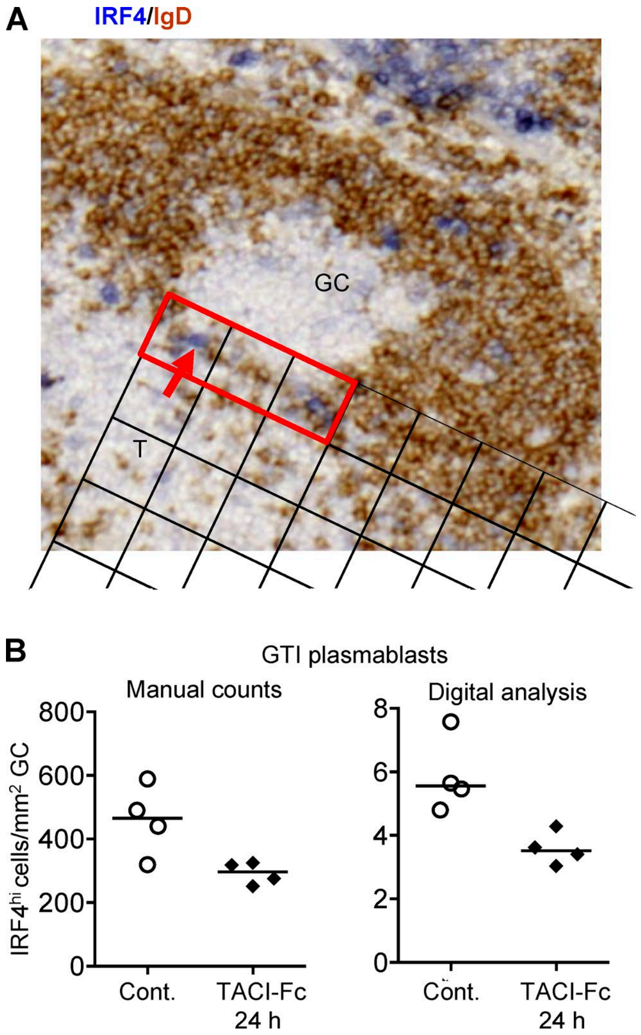

Figure S4. **Manual stereological analysis of tissue sections and its validation by digital image analysis. (A)** Manual quantification of IRF4^{hi} at the GC-T zone interface. A 100-field 1 cm^2 ocular counting grid was moved over the center of the GTI projected using a 25× microscope lens. Using a 25× lens the width of one field is 40 µm. Cells were counted if they fell into this 40-µm wide band. GC area was quantified using point counting and the same counting grid (one intercept corresponds to 1,600 µm^2). Tissues were blinded and randomized before quantification. Some tissues were analyzed by an independent examiner with good reproducibility. **(B)** To test the reliability of the manual counting method, a semiautomated analysis method was designed to measure blue staining area within the GTI from whole spleen sections. In brief, spleen sections immunoenzymatically stained for IRF4 and IgD were scanned using a 10× lens of an AxioScan microscope scanner (Zeiss). Using Fiji (Link et al., 2007), brown and blue colors were separated by color deconvolution. Follicular areas were segmented from the brown channel smoothed using a median filter. The GTI was segmented by manually drawing a line along the GC–T zone border. The area of the adjacent GC was measured as the IgD negative compartment enclosed by IgD staining and the line defining the GTI. The area covered by IRF4 staining was measured by widening the line defining the GTI to 40 µm, and measuring segmented blue staining area within this band in the blue channel. This was done for all GCs on a section, and data were exported into Excel and Prism for statistical analysis.

Table S1. **Sequence of primers and probes used for qRT-PCR**

Primer	Forward primer sequence (5′–3′)	Reverse primer sequence (5′–3′)	Probe sequence (5′–3′)
IRF4	GGAGGACGCTGCCCTCTT	TCTGGCTTGTCGATCCCTTCT	AGGCTTGGGCATTGTTTAAAGGCAAGTTC
Blimp1	CAAGAATGCCAACAGGAAGTATTTT	CCATCAATGAAGTGGTGGAACTC	TCTCTGGAATAGATCCGCCA
XBP-1	GGTGCAGGCCCAGTTGTC	CAAAAGGATATCAGACTCAGAATCTGAA	TCCCATGGACTCTGACACTGTTGCCTC
AID	GTCCGGCTAACCAGACAACTTC	GCTTTCAAAATCCCAACATACGA	GCTTTCAAAATCCCAACATACGA
Bcl-6	CAGACGCACAGTGACAAACCA	ACTGCGCTCCACAAATGTTACA	ACTGCGCTCCACAAATGTTACA
PAX 5	GCTGTTGGCAGAGCGAGTCT	AGCTGGGACCGGCTGATT	ACACTGTGCCCAGCGTCAGCTCC
IgG1 ST	CGAGAAGCCTGAGGAATGTGT	GGAGTTAGTTTGGGCAGCAGAT	TGGTTCTCTCAACCTGTAGTCCATGCCA
Iμ-Cγ1	TCTGGACCTCTCCGAAACCA	CCAGGGTCACCATGGAGTTAGT	TGCCAAAACGACACCCCCATCTGT
J Chain	GTCCTGGCCATTTTTGTTAAGG	ACATGCATTTGTTGTCAGCAAGA	TGGTCGCTTCGTCGTCACCTGTTACA
CXCR5	GCTCTGCACAAGATCAATTTCTACTG	CCGTGCAGGTGATGTGGAT	CCATCGTCCATGCTGTTCACGCC
CXCL12	CAAGCATCTGAAAATCCTCAACAC	CACTTTAATTTCGGGTCAATGCA	TGCACGGCTGAAGAACAACAACAGACAA
IL4	GATCATCGGCATTTTGAACGA	AGGACGTTTGGCACATCCAT	TGCATGGCGTCCCTTCTCCTGTG
IL21	ACACCCAAAGAATTCCTAGAAAGACTAA	TGCATTCGTGAGCGTCTATAGTG	AGCATCTCTCCTAGAACACATAGGACCCGAAGAT
CXCR4	TGCTCCGGTAACCACCAC	CCAGAACCCACTTCTTCAGAGTAG	TAGAGCGAGTGTTGCCATGGAACC
CXCL13	ACATCATAGATCGGATTCAAGTTACG	TTCACACATATACTTTCTTCATCTTGGT	CCTGGGAATGGCTGCCCCAAA
BAFF	GAAGTGTGCCATGTGAGTTATGAGA	TCACCCAAGGCAAAAAGCA	TCCTTTGCCAACACGCACCGC
BAFF-R	ACTTCAGAAGGAGTCCAGCAAGAG	CAGGTAGGAGCTGAGGCATGAG	CCCTGGAAAATGTCTTTGTACCCTCCTCA
APRIL	CGAGTCTGGGACACTGGAATTT	AGATACCACCTGACCCATTGTGA	CTGCTCTATAGTCAGGTCCTGTTTCATGATGTGAC
TACI	CATTCTGCCCCAAAGATCAGTAC	TGCTCTTTTCGGCAATTGATG	CAGCCAGAGGAGCCAGCGCAC
BCMA	TCCAACCCTCCTGCAACCT	CGTGTACGTCCCTTTCACTGAA	TCAGCCTTACTGTGATCCAAGCGTGACC
CCL19	CCTTCCGCTACCTTCTTAATGAAG	ACAGAGCTGATAGCCCCTTAGTGT	TGCAGGGTGCCTGC
CCL21	TCCCGGCAATCCTGTTCTC	TTCTGCACCCAGCCTTCCT	CCCCGGAAGCACTCTAAGCCTGAGCTAT
β2m	CTGCAGAGTTAAGCATGCCAGTAT	ATCACATGTCTCGATCCCAGTAGA	CGAGCCCAAGACC

All primers and probes are from Eurofins Genomics (Ebersberg Germany). CD3e (Mm00599683_m1), CD19 (Mm00515420_m1), and IL21R (Mm00600319_m1) are TaqMan gene expression assay (Thermo Fisher).

References

Fletcher, A.L., D. Malhotra, and S.J. Turley. 2011. Lymph node stroma broaden the peripheral tolerance paradigm. *Trends Immunol.* 32:12–18. https://doi.org/10.1016/j.it.2010.11.002

Link, A., T.K. Vogt, S. Favre, M.R. Britschgi, H. Acha-Orbea, B. Hinz, J.G. Cyster, and S.A. Luther. 2007. Fibroblastic reticular cells in lymph nodes regulate the homeostasis of naive T cells. *Nat. Immunol.* 8:1255–1265. https://doi.org/10.1038/ni1513
